# Beyond Tuberculosis: Unlocking the Antifungal Potential of Novel Isoniazid Derivatives against Non-*Candida albicans* Candida Species

**DOI:** 10.1021/acsomega.6c00859

**Published:** 2026-09-11

**Authors:** Tebogo M. L. Mokoto, Itumeleng B. Setshedi

**Affiliations:** † Biotechnology Niche Area, College of Science, Engineering and Technology, 274780 University of South Africa, Johannesburg 1709, Gauteng, South Africa; ‡ Department of Life and Consumer Science, College of Agriculture and Environmental Science, University of South Africa, Johannesburg 1709, Gauteng, South Africa

## Abstract

The increasing prevalence of drug-resistant non-*Candida* *albicans* *Candida* (NCAC) species has created an urgent need for novel antifungal agents with improved efficacy and selectivity. In this study, eight modified isoniazid derivatives were synthesized through hydrazone formation and structurally characterized using single-crystal X-ray diffraction (SC-XRD), Fourier transform infrared (FTIR) spectroscopy, Raman spectroscopy and nuclear magnetic resonance (NMR) analysis. The antioxidant potential of the derivatives was evaluated using the DPPH radical scavenging assay, while antifungal activity was assessed against *Candida auris*, *Candida glabrata*, and *Candida parapsilosis* using minimum inhibitory concentration (MIC) assays. Several derivatives demonstrated enhanced antifungal activity relative to the parent compound, with Compounds II, V, and VI exhibiting the strongest activity against *C. auris* (MIC = 15.6 μg/mL). Compound II also displayed broad-spectrum activity across all tested *Candida* species. Flow cytometry using propidium iodide staining revealed increased membrane permeability and loss of cellular integrity following treatment, supporting membrane disruption as a potential contributor to antifungal activity. Antioxidant evaluation showed that Compound VII (17.61 μg/mL) possessed the strongest radical scavenging activity among the derivatives, with an IC_50_ value approaching that of ascorbic acid (9.7 μg/mL). Cytotoxicity assessment using Vero cells demonstrated concentration-dependent effects, with variable LC_50_ values observed across the series. Real-time cell analysis (RTCA) further revealed dynamic differences in cellular responses following compound exposure, with Compound III exhibiting comparatively lower effects on mammalian cell viability. Structure–activity relationship analysis suggested that phenolic functionalities, lipophilic substituents and hydrazone-linked modifications contribute to both antifungal and antioxidant behaviour.

## Graphical abstract

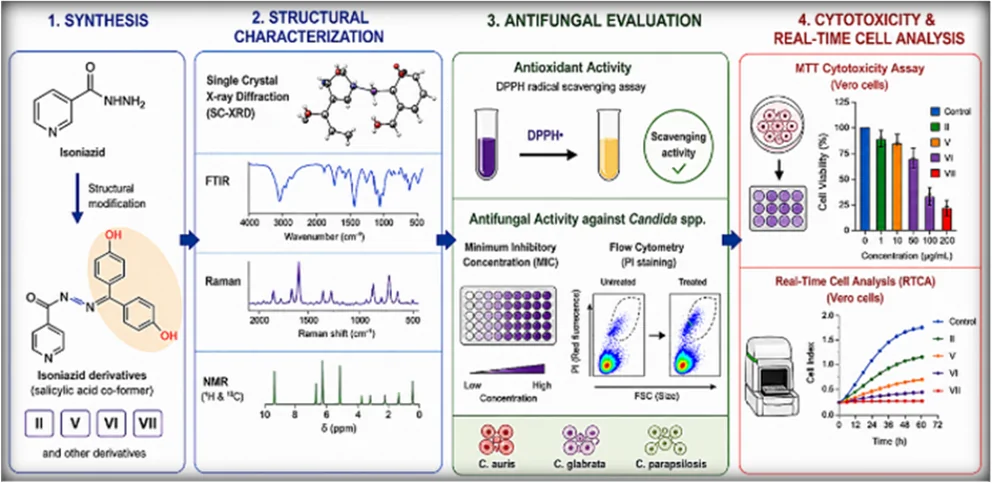

## Introduction

Candidiasis is a major cause of invasive fungal disease worldwide and is associated with significant morbidity and mortality, particularly in immunocompromised and critically ill patients. Although *Candida* species commonly exist as commensals, disruption of host immunity or microbiota can lead to opportunistic infection. Of the approximately 350 identified *Candida* species, only a limited number are pathogenic to humans; however, their clinical impact is substantial.^1,2^

Historically, *Candida albicans* has been the predominant cause of candidiasis. In recent years, however, a marked epidemiological shift toward non-*Candida albicans* *Candida* (NCAC) species has been observed, particularly *Candida auris*, *Candida glabrata*, and *Candida parapsilosis*.^3^ These species are increasingly implicated in invasive candidiasis, including candidemia and nosocomial bloodstream infections, and pose a serious global health concern due to their intrinsic or acquired resistance to multiple antifungal agents.^4,5^ *Candida auris* has emerged as a high-priority pathogen because of its rapid nosocomial transmission, environmental persistence, and frequent resistance to azoles, echinocandins, and polyenes. Similarly, *C. glabrata* exhibits reduced susceptibility to azoles, while *C. parapsilosis* is associated with biofilm-mediated infections and decreased responsiveness to echinocandins, especially in catheter-related diseases.^6^

The clinical management of NCAC infections is further complicated by biofilm formation, which confers enhanced tolerance to antifungal therapy and contributes to the persistence and recurrence of infections.^7^ However, the effects of novel compounds on these resistant phenotypes remain underexplored. These factors underscore the urgent need for novel or alternative antifungal strategies that are effective against NCAC species and can overcome current resistance mechanisms. Drug repurposing represents a pragmatic and accelerated approach to antifungal discovery by identifying new therapeutic uses for existing drugs with established safety profiles.^8,9^

Within this context, isonicotinic acid hydrazide (Isoniazid, INH), a first line antitubercular agent, has emerged as a promising scaffold for antimicrobial repurposing. Its suitability as a repurposing candidate lies in its favorable physicochemical and structural features. Beyond its well-established role in tuberculosis treatment, isoniazid and its derivatives have demonstrated broad-spectrum antimicrobial activity, including efficacy against *Candida albicans* and other fungal and bacterial pathogens.^10^

The molecule possesses a hydrazide functional group capable of participating in hydrogen bonding and metal coordination, as well as a pyridine ring that contributes to lipophilicity and membrane permeability.^11^ These properties support interactions with diverse biological targets, including enzymes and nucleic acids, and facilitate intracellular accumulation. Moreover, the chemical simplicity of isoniazid allows for straightforward structural modification, enabling the generation of derivatives with enhanced antifungal potency, selectivity and pharmacokinetic profiles.^12^

Overall, the structural adaptability and bioactive potential position isoniazid as a compelling candidate for the development of novel antifungal agents aimed at combating drug-resistant non-*Candida albicans* *Candida* (NCAC) infections, where innovative therapeutic strategies remain urgently needed.

## Results

### Synthesis and Characterization of Isoniazid Derivatives

Synthesised isoniazid derivatives retain the amide group alongside a newly formed imine functionality. In this modification, the primary amine (−NH_2_) of isoniazid reacted with a carbonyl compound through a Schiff base condensation, resulting in the formation of a C=N (imine) bond while eliminating water. The substituent labelled as R^1^ is introduced at the imine nitrogen, thereby modifying the original isoniazid framework (Figure 1).

**Figure 1:**
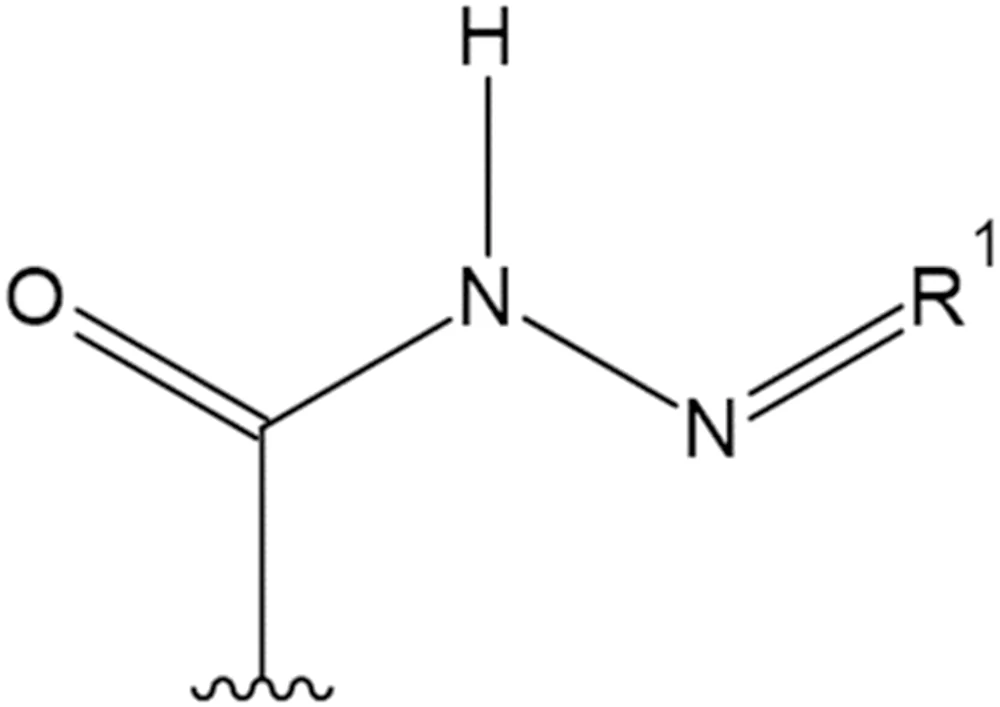
Structural modifications of isoniazid were used to evaluate the antifungal activity against Candida spp. (ChemSketch Version 2023.2.4).

Structurally, all compounds retain the pyridine-4-carbohydrazide core of isoniazid, with substitution occurring at the terminal hydrazide nitrogen through condensation with substituted aldehydes. This modification introduces an azomethine (–C=N–) linkage, which is a defining feature across Compounds I–VIII and plays a key role in modulating both electronic properties and intermolecular interactions (Table 1).

**Table 1:**
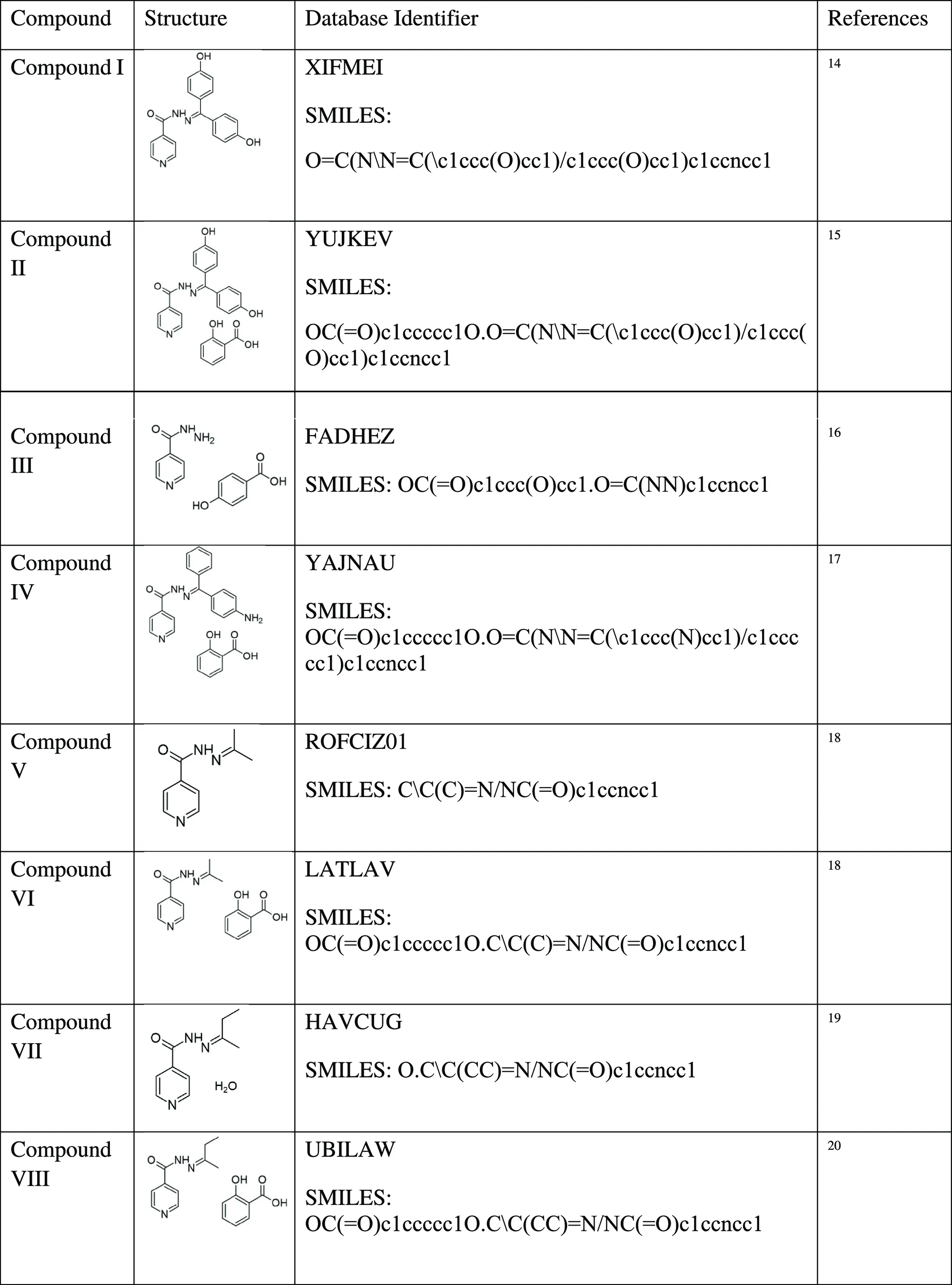
Chemical Structures, Database Identifiers (Cambridge Structural Database, CSD), SMILES and Corresponding Literature References for Isoniazid-Derived Compounds (I–VIII) Used in This Study

A common structural framework was observed across the series of isoniazid derivatives and their respective co-crystals. These consist of three key components: the pyridine ring, the hydrazone linkage, and the substituted aromatic ring. The pyridine nucleus, conserved in all derivatives, provides a hydrogen-bond acceptor site through its nitrogen ring and contributes to molecular planarity, which is central to the supramolecular assembly observed in the co-crystals. The hydrazone linkage (−CONH–N=CH−), formed via condensation, introduces conjugation between the pyridine core and the appended aromatic ring. This linkage with the C=N double bond enhances rigidity and facilitates π-electron delocalization across the molecule.^13^ Structural variations arise primarily from the substituents on the aromatic ring (R_1_). These include phenolic hydroxyl groups, methoxy groups, and alkyl or substituted aromatic functionalities. These substituents significantly influence electronic distribution, hydrogen-bonding potential, and the overall supramolecular architecture.

Co-crystal formation seen in Compounds II, IV, VI, and VIII, respectively, further expands the hydrogen-bonding landscape. The carboxylic acid co-formers, such as salicylic acid, engage in O–H···N interactions with the pyridine and hydrazone functionalities on the isoniazid molecule. The phenolic co-formers establish cooperative hydrogen-bonding networks with the pyridine nitrogen and hydrazone moiety. These interactions stabilize the lattice, enhance packing efficiency, and modulate physicochemical properties such as polarity and solubility. The systematic variation in R_1_ substituents combined with co-crystal formation underscores the versatility of the isoniazid scaffold in accommodating diverse supramolecular synthons, highlighting co-crystal engineering as a powerful strategy for tuning molecular recognition, stability, and potential pharmaceutical relevance.

### Fourier-Transform Infrared Resonance and Raman Spectroscopy

The structural evolution from parent isoniazid to Compounds I–VIII was validated using a combination of single-crystal X-ray diffraction (SCXRD), FTIR, and Raman spectroscopy (Table S1). The complementary nature of FTIR and Raman spectroscopy enabled detailed evaluation of both polar functional groups and aromatic or backbone vibrations across the series.

The FTIR–Raman overlay of Compound I (Figure S1) confirmed formation of the hydrazone derivative. FTIR spectra exhibited characteristic polar features, including broad O–H/N–H stretching (3300–3400 cm^–1^), a prominent amide I band (1650–1700 cm^–1^), and a defined amide II vibration (1558 cm^–1^). Raman spectra complemented these findings by highlighting hydrazone C=N stretching (1620 cm^–1^) and aromatic ring vibrations (1000–1200 cm^–1^). Together with SCXRD, these data confirm formation of the hydrazone linkage and associated hydrogen-bonding interactions. For Compound II (Figure S2), significant spectral changes were observed consistent with co-crystal formation with salicylic acid. FTIR spectra showed broadening of the O–H region and perturbation of the carbonyl band, while Raman spectra revealed enhanced aromatic ring vibrations (1600 cm^–1^) and COOH-related modes (1400 cm^–1^). These features, supported by SCXRD, confirm formation of a hydrogen-bonded co-crystal lattice.

Compound III (Figure S3) displayed reduced spectral perturbation relative to the co-crystals. FTIR showed less pronounced broadening in the O–H/N–H region and a weaker carbonyl band, while Raman spectra indicated moderate hydrazone C=N (1620 cm^–1^) and aromatic contributions. These results, in agreement with SCXRD, confirm Compound III as an independent hydrazone derivative lacking co-crystal integration. In contrast, Compound IV (Figure S4) exhibited clear signatures of co-crystal formation. FTIR spectra showed broadened O–H stretching and shifted carbonyl absorption, while Raman spectra emphasized aromatic ring vibrations (1600 cm^–1^) and COOH deformation (1400 cm^–1^). These complementary features, together with SCXRD, confirm incorporation of salicylic acid and stabilization via hydrogen bonding and π–π interactions.

A similar trend was observed for Compound V (Figure S5), which displayed features consistent with a single-component hydrazone derivative. FTIR indicated reduced hydrogen-bonding effects, while Raman spectra highlighted contributions from the isopropyl substituent (C–H deformation 1450 cm^–1^), alongside hydrazone C=N and aromatic modes. SCXRD confirmed the absence of co-crystal formation. Upon co-crystallization, Compound VI (Figure S6) showed broadened O–H stretching, enhanced carbonyl intensity, and increased aromatic contributions in both FTIR and Raman spectra, consistent with salicylic acid incorporation. These findings were supported by SCXRD, confirming formation of a hydrogen-bonded co-crystal.

Compound VII (Figure S7) was identified as a hydrated hydrazone derivative. FTIR spectra showed broadened O–H/N–H stretching attributable to lattice water, while Raman spectra indicated aromatic ring vibrations (1600 cm^–1^), hydrazone C=N (1620 cm^–1^), and water bending modes (1640 cm^–1^). SCXRD confirmed incorporation of water molecules, contributing to lattice stabilization. Finally, Compound VIII (Figure S8) exhibited the most pronounced spectral changes. FTIR revealed extensive broadening in the O–H region and further perturbation of the carbonyl band, while Raman spectra showed strong aromatic and COOH-related vibrations. These results, supported by SCXRD, confirm co-crystal formation involving both salicylic acid and lattice water, indicating enhanced intermolecular stabilization.

The FTIR-Raman overlay of the parent isoniazid (Figure S9) provided the reference vibrational profile for the series. FTIR spectra showed characteristic hydrazide features, including N–H stretching (3300–3400 cm^–1^), a strong carbonyl band (1650–1700 cm^–1^), and a distinct amide II vibration (1558 cm^–1^). Raman spectra highlighted pyridine ring vibrations, including aromatic C=C/C=N stretching (1600–1620 cm^–1^) and ring breathing modes (1000–1200 cm^–1^). Notably, no hydrazone C=N stretching is present in the parent compound. Comparison across the series demonstrates that co-crystal formation (Compounds II, IV, VI, and VIII) is associated with O–H/N–H broadening, carbonyl perturbation and enhanced aromatic contributions. In contrast, independent derivatives (Compounds I, III, V, and VII) retain more defined vibrational features with hydrazone C=N bands arising from structural modification.

### Nuclear Magnetic Resonance

#### ^1^H NMR Spectral Analysis

The ^1^H NMR spectrum of isoniazid (D_2_O) (Table S2.1) showed the expected pyridine resonances as two multiplets at δ 8.67–8.61 ppm (2H, H-2/H-6) and δ 7.69–7.63 ppm (2H, H-3/H-5), consistent with the electron-withdrawing effect of the ring nitrogen. A broad signal at δ 4.7–4.9 ppm was assigned to HOD, while exchangeable hydrazide NH/NH_2_ protons were not observed due to rapid proton exchange in D_2_O.

Compound I (Figure S10 and Table S2.2) displayed pyridine signals at δ 8.50–8.69 ppm (2H) and overlapping aromatic resonances at δ 7.7–7.9 ppm, attributed to pyridine and *p*-hydroxyphenyl protons. Additional multiplets at δ 6.9–7.0 ppm correspond to the para-substituted phenyl ring. Exchangeable OH/NH protons were not observed, and a residual HOD signal appeared at δ 4.8 ppm, confirming hydrazone formation. Compound II (Figure S12 and Table S2.3) exhibited a downfield pyridine signal at δ 8.60 ppm (2H, H-2/H-6) and overlapping aromatic resonances at δ 7.79 ppm, assigned to pyridine and salicylate protons. Broad multiplets between δ 7.55–6.64 ppm correspond to salicylate and p-hydroxyphenyl aromatic environments. A weak broad signal at δ 10.16 ppm may indicate an exchangeable proton, although assignment is uncertain in D_2_O. Residual solvent signals (δ 3.36–1.92 ppm) were also observed.

Compound III (Figure S13 and Table S2.4) showed pyridine resonances at δ 8.6 ppm (2H) and overlapping aromatic signals at δ 7.8–7.5 ppm, with additional multiplets at δ 6.9–6.7 ppm assigned to p-hydroxybenzoate protons. A broad HOD signal appeared at δ 4.8 ppm, while exchangeable protons were not observed. Compound IV (Figure S15 and Table S2.5) displayed aromatic resonances between δ 8.69–6.92 ppm, with pyridine signals at δ 8.69–8.59 ppm (2H). Overlapping multiplets at δ 7.83–7.72 ppm and δ 7.51–6.92 ppm were assigned to salicylate, phenyl, and aminophenyl protons. A residual solvent signal at δ 3.36 ppm and HOD at δ 4.8 ppm were observed, while exchangeable protons were absent.

Compound V (Figure S17 and Table S2.6) showed pyridine resonances at δ 8.58–8.52 ppm (2H) and δ 7.77–7.62 ppm (2H). Two singlets at δ 2.05 ppm and δ 1.88 ppm correspond to methyl groups on the C=N–C­(CH_3_)_2_ fragment. Residual acetone (δ 2.23 ppm), HOD (δ 4.81 ppm), and TMS (δ 0.00 ppm) were also observed. Compound VI (Figure S19 and Table S2.7) exhibited pyridine signals at δ 8.80–8.72 ppm and δ 7.98–7.90 ppm, with extensive salicylate resonances between δ 7.92–6.84 ppm. Methyl signals of the hydrazone moiety appeared at δ 2.22–2.06 ppm. A broad HOD signal was observed at δ 4.90 ppm.

Compound VII (Figure S21 and Table S2.8) showed pyridine resonances at δ 8.67–8.61 ppm and δ 7.69–7.63 ppm, with no definitive hydrazone alkyl signals. A broad HOD peak appeared at δ 4.68 ppm, and minor low-intensity signals were attributed to trace impurities. Compound VIII (Figure S23 and Table S2.9) displayed pyridine signals at δ 8.84–8.71 ppm and δ 7.90–7.84 ppm, with salicylate aromatic resonances at δ 7.78 ppm, δ 7.44 ppm, and δ 6.99–6.87 ppm, consistent with a substituted pyridine–salicylate hydrazone framework.

#### ^13^C NMR Spectral Analysis

The ^13^C NMR spectrum of isoniazid (Table S3.1) displayed a characteristic hydrazide carbonyl resonance at δ 166.62 ppm, assigned to the CONH–NH_2_ carbonyl carbon. Signals at δ 149.37 ppm correspond to the pyridine C-2/C-6 carbons adjacent to the ring nitrogen, while the resonance at δ 141.07 ppm was assigned to the pyridine ipso carbon (C-4). The pyridine CH carbons (C-3/C-5) appeared at δ 121.47 ppm.

Compound I (Figure S11 and Table S3.2) exhibited resonances consistent with the proposed *p*-hydroxyphenyl hydrazone framework. The signal at δ 159.63 ppm was assigned to the phenolic C–OH carbon, with possible overlap from conjugated C=N/C=O environments. Pyridine C-2/C-6 carbons appeared at δ 148.66 ppm, while aromatic CH carbons of the *p*-hydroxyphenyl ring were observed at δ 131.73 ppm and δ 115.06 ppm. Pyridine C-3/C-5 carbons resonated at δ 121.96 ppm. For Compound II, the ^13^C NMR spectrum exhibited significant noise and poor signal resolution, preventing reliable assignment of individual carbon resonances. Nevertheless, the obtained data were broadly consistent with the proposed structure and supported by complementary SC-XRD, FTIR, Raman, and ^1^H NMR analyses.

Compound III (Figure S14 and Table S3.3) showed pyridine C-2/C-6 resonances at δ 149.19 ppm, consistent with deshielding by the pyridine nitrogen. Aromatic carbons of the p-hydroxybenzoate moiety appeared at δ 129.41 ppm, while overlapping pyridine/aryl CH signals were observed at δ 121.74 ppm. The ^13^C NMR spectrum of Compound IV (Figure S16 and Table S3.4) displayed aromatic carbon resonances between δ 133.94–116.25 ppm, corresponding to phenyl, aminophenyl and salicylate environments. Signals at δ 133.94 ppm and δ 130.45 ppm were assigned to aromatic carbons and CH groups of the phenyl/salicylate rings, while resonances at δ 119.33 ppm and δ 116.25 ppm correspond to aminophenyl/salicylate aromatic CH carbons.

Compound V (Figure S18 and Table S3.5) displayed characteristic carbonyl and hydrazone resonances at δ 163.57 ppm and δ 162.87 ppm, assigned to the hydrazide C=O and imine (C=N) carbons, respectively. Pyridine carbons adjacent to the ring nitrogen (C-2/C-6) appeared at δ 148.83 and 148.70 ppm, while pyridine CH carbons (C-3/C-5) resonated at δ 122.11 and 121.33 ppm. The methyl carbons of the C=N–C­(CH_3_)_2_ fragment were observed at δ 23.85 ppm and δ 17.15 ppm, consistent with the proposed hydrazone structure. Compound VI (Figure S20 and Table S3.6) exhibited resonances characteristic of the salicylate hydrazone framework. Signals at δ 174.75 ppm and δ 169.29 ppm were assigned to the salicylate and hydrazide carbonyl carbons, respectively, while the imine carbon resonance appeared at δ 165.62 ppm. The phenolic salicylate carbon (C–OH) was observed at δ 159.51 ppm. Pyridine carbons adjacent to nitrogen resonated at δ 146.64 and 146.57 ppm, with additional quaternary aromatic carbons appearing at δ 144.02 and 143.52 ppm. Aromatic CH carbons from the salicylate and pyridine rings were distributed between δ 134.14–116.24 ppm. The hydrazone methyl carbons appeared at δ 24.07 ppm and δ 17.94 ppm. Residual acetone signals at δ 215.24 ppm and δ 30.15 ppm were attributed to solvent impurities and not to the target compound.

The ^13^C NMR spectrum of Compound VII (Figure S22 and Table S3.7) closely resembled that of isoniazid, with resonances at δ 166.62 ppm (hydrazide carbonyl), δ 149.37 ppm (Py-C2/C6), δ 141.07 ppm (Py-C4), and δ 121.47 ppm (Py-C3/C5). However, the expected alkyl carbon signals associated with the hydrazone fragment below δ 50 ppm were not observed, preventing definitive confirmation of the proposed alkyl-substituted hydrazone moiety by ^13^C NMR alone. Compound VIII (Figure S24 and Table S3.8) showed characteristic salicylate and pyridine resonances, including the salicylate carbonyl carbon at δ 175.05 ppm and a hydrazide/C=N resonance at δ 166.10 ppm. The phenolic salicylate carbon appeared at δ 159.54 ppm, while pyridine carbons adjacent to nitrogen resonated at δ 147.09 ppm. Additional aromatic carbons were observed between δ 143.22–116.27 ppm, corresponding to pyridine and salicylate aromatic environments. Although the aromatic and carbonyl resonances support the proposed structure, the expected alkyl carbon signals for the butanone-derived hydrazone fragment below δ 50 ppm were not clearly resolved in the spectrum.

### Antioxidant Activity Assay

To assess the redox and antioxidant potential of the synthesised compounds, DPPH (2,2-diphenyl-1-picrylhydrazyl) radical scavenging activity was evaluated.^21^ Initially, DPPH solution appears as a deep violet colour; however, upon neutralization to DPPH-H, it transitions to pale yellow or becomes colourless. This colour change signifies that the tested derivative possesses hydrogen-donating capabilities, indicative of its antioxidant activity.^22^ IC_50_ values were determined by fitting the concentration–response data to a nonlinear regression model using a four-parameter logistic (4PL) equation. The goodness of fit was assessed using *R* ^2^ values, with values greater than 0.9 considered acceptable (Figure 2).

**Figure 2:**
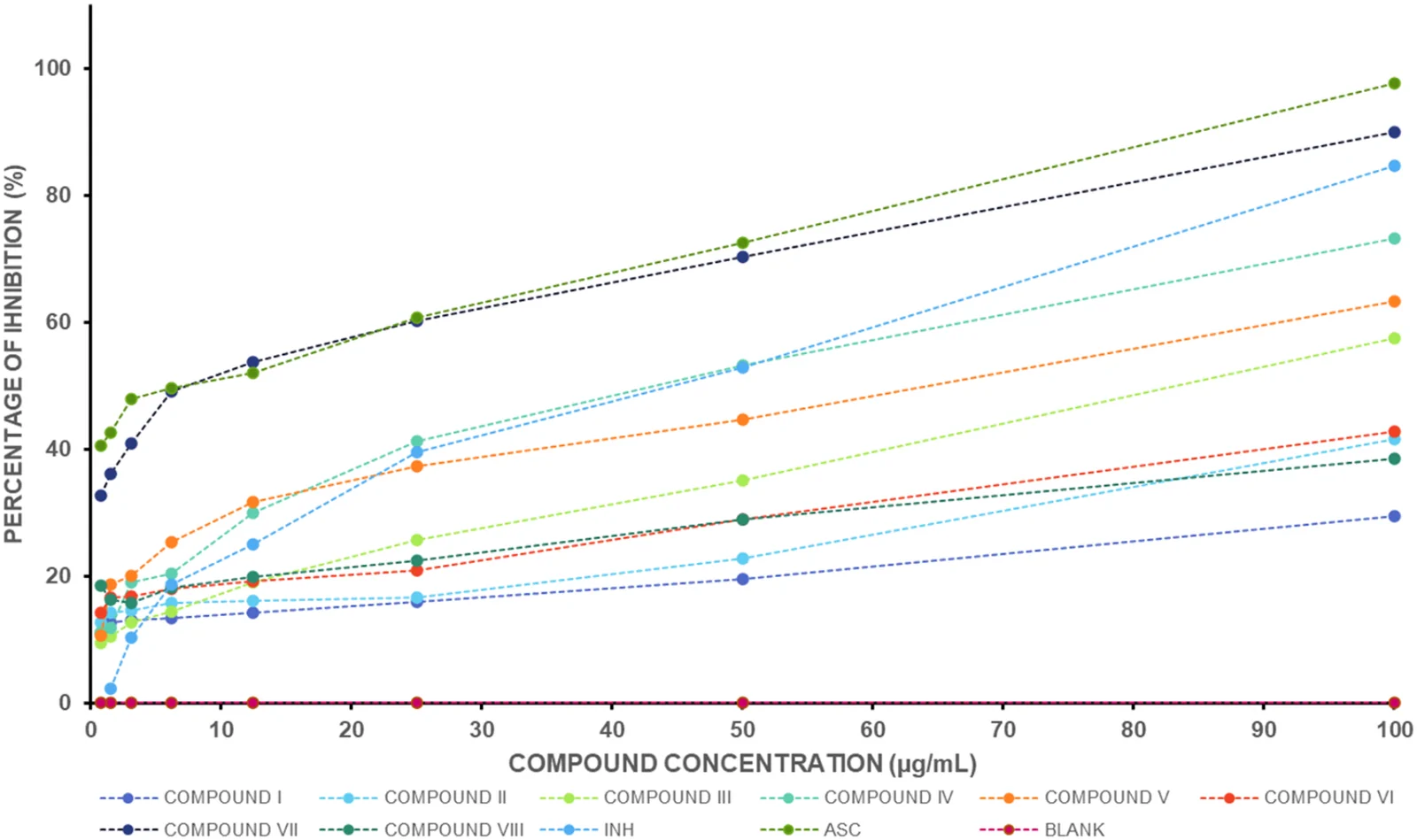
Half-maximal inhibitory concentration (IC_50_) plots showing the dose-dependent antioxidant inhibition of the compounds. Data were fitted using a four-parameter logistic (4PL) model (GraphPad Prism Version 5.03).

Antioxidant activity of isoniazid (INH) and its derivatives (Compounds I–VIII) was evaluated using DPPH radical scavenging assay, and the results are summarized in Table 2. The compounds exhibited a broad range of activity, with IC_50_ values ranging from 17.61 to 222.27 μg/mL, indicating that structural modification of the INH scaffold significantly influences radical-scavenging capacity. This is due to the parent compound Isoniazid (INH) exhibiting moderate activity (IC_50_ = 50.67 ± 0.03 μg/mL, 369.6 ± 0.2 μM).

**Table 2:** DPPH Assay IC_50_ Values for Modified Isoniazid Derivatives and Ascorbic Acid Measured in μg/mL and Converted to μM^a^,^b^

Compounds	IC_50_ value (μg/mL)	IC_50_ value (μM)
Compound I	222.27 ± 0.02	667.0 ± 0.1
Compound II	139.49 ± 0.01	296.0 ± 0.1
Compound III	82.15 ± 0.01	298.4 ± 0.1
Compound IV	53.54 ± 0.01	117.8 ± 0.1
Compound V	64.84 ± 0.02	365.9 ± 0.1
Compound VI	127.47 ± 0.01	404.2 ± 0.1
Compound VII	17.61 ± 0.01	92.1 ± 0.1
Compound VIII	149.48 ± 0.01	453.7 ± 0.1
Isoniazid (INH)	50.67 ± 0.03	369.6 ± 0.2
Ascorbic acid (ASC)	9.70 ± 0.01	55.1 ± 0.1

a Values are expressed as mean ± SD (*n* = 3).

b *p* < 0.05.

Among the derivatives, Compound VII demonstrated the most potent activity (IC_50_ = 17.61 ± 0.01 μg/mL; 92.1 ± 0.1 μM), approaching that of the reference antioxidant Ascorbic acid (IC_50_ = 9.70 ± 0.01 μg/mL; 55.1 ± 0.1 μM). In contrast, Compound I exhibited the weakest activity (IC_50_ = 222.27 ± 0.02 μg/mL; 667.0 ± 0.1 μM), suggesting minimal contribution of its substituent toward radical stabilisation.

A clear structure-activity relationship (SAR) trend is observed across the series of derivatives bearing electron-donating and hydrogen bond–active substituents. The phenolic −OH groups displayed enhanced antioxidant activity. This is evident in Compounds III (82.15 ± 0.01 μg/mL; 298.4 ± 0.1 μM), IV (53.54 ± 0.01 μg/mL; 117.8 ± 0.1 μM), and especially VII (17.61 ± 0.01 μg/mL; 92.1 ± 0.1 μM), where increased activity can be attributed to the ability of these groups to donate hydrogen atoms to neutralise DPPH radicals and stabilise the resulting radical species through resonance delocalisation.

In contrast, derivatives incorporating less reactive or sterically hindered substituents, such as Compounds II, VI, and VIII, with IC_50_ values from 127 to 149 μg/mL, exhibited reduced activity. These results are likely due to limited hydrogen-donating capacity and decreased accessibility of reactive sites. Similarly, compounds with increased lipophilic character, such as Compound V with IC_50_ values of 64.84 ± 0.02 μg/mL (365.9 ± 0.1 μM), showed moderate activity. This suggests that while lipophilicity may enhance membrane interaction, it does not directly correlate with radical scavenging efficiency.

Isoniazid, as the parent drug, demonstrated concentrations comparable to derivatives such as Compound IV but inferior to the most active analogue, Compound VII. This indicates that the introduction of appropriately positioned functional groups can significantly enhance antioxidant performance beyond the parent scaffold.^23^

Antioxidant activity in this series is largely determined by electron-donating substituents positioned for hydrogen atom transfer and the degree of conjugation within the hydrazone linkage. Compounds that balance planarity, conjugation, and hydrogen-donating capacity show stronger radical scavenging. Variations in IC_50_ values reflect the influence of electron-donating or electron-withdrawing groups on radical neutralization. Compound VII, with its low IC_50_, is the most promising candidate for further investigation.

### Minimum Inhibitory Concentration

Based on their chemical properties, the synthesised derivatives were evaluated for antifungal activity against clinically relevant non-albicans Candida (NCAC) species using MIC determination. The modified isoniazid derivatives were tested at varying concentrations against *Candida auris*, *Candida glabrata*, and *Candida parapsilosis*, with amphotericin B and isoniazid included as reference controls (Table 3).

**Table 3:** Minimum Inhibitory Concentration (μg/mL) of Modified Isoniazid Derivatives against *Candida* Species and Positive Control, Amphotericin B

	*Candida auris*	*Candida parapsilosis*	*Candida glabrata*
Compound I	31.2	250	31.2
Compound II	15.6	31.2	62.5
Compound III	125	250	125
Compound IV	>250	250	250
Compound V	15.6	62.5	250
Compound VI	15.6	62.5	250
Compound VII	31.2	62.5	250
Compound VIII	31.2	62.5	250
Isoniazid	>500	>500	>500
Amphotericin B	1.25	1.25	1.25

Against *C. auris*, Compounds II, V, and VI exhibited the strongest activity, each with MIC values of 15.6 μg/mL. Compounds I and VII demonstrated moderate activity at 31.2 μg/mL, while Compounds III and IV showed weaker inhibition with concentrations of 125 μg/mL and >250 μg/mL, respectively. For *C. parapsilosis*, Compound II again displayed the most potent activity (31.2 μg/mL). Compounds V, VI, VII, and VIII showed moderate efficacy with MIC values of 62.5 μg/mL. Compounds I, III, and IV were less active with concentrations greater than 250 μg/mL. In *C. glabrata*, Compound I was the most effective derivative with a concentration of 31.2 μg/mL. This was followed by Compound II with an MIC of 62.5 μg/mL. Other derivatives, including Compounds V–VIII, exhibited limited activity with MIC values greater than or equivalent to 250 μg/mL.

Overall, Compound II consistently demonstrated strong antifungal activity across all three Candida species, outperforming isoniazid under the tested conditions. Compounds V and VI were effective against *C. auris* and *C. parapsilosis* but lacked activity against *C. glabrata*. Derivatives with MIC values greater than 250 μg/mL, such as Compounds III and IV, were considered to have limited antifungal potential.

### Flow Cytometry

To investigate the potential mechanism underlying antifungal activity, compounds demonstrating inhibitory effects were further assessed for their ability to induce cellular disruption in *Candida* cells. *Candida* cells were stained with propidium iodide (PI) to assess membrane integrity, where PI-positive populations represent non-viable cells. Flow cytometric analysis was performed using forward scatter (FSC-H) and side scatter (SSC-H) parameters to evaluate cell size and internal complexity, respectively. Cell populations were gated on FSC/SSC plots to exclude debris and aggregates, and viability was determined based on PI fluorescence intensity. Data were analyzed using BD FACSDiva software (Version 8.0.1).

This experiment aimed to evaluate the *in vitro* antifungal effects of the tested compounds following 24 h exposure at concentrations based on their minimum inhibitory concentrations (MICs). Amphotericin B was included as a reference control. Following treatment, shifts in FSC/SSC profiles and increased PI staining were observed, indicating changes in cell morphology and loss of membrane integrity. These findings are consistent with treatment-induced cellular damage and cell death. However, because only PI staining was employed, the data do not distinguish between apoptotic and necrotic pathways, and no specific mode of cell death can be inferred.

Flow cytometry assays were performed at concentrations ≤125 μg/mL to maintain biological relevance and to minimize non-specific cytotoxic effects associated with higher doses, which may result in extensive cell lysis and obscure mechanistic interpretation.^24^ Flow cytometry is most informative at concentrations near the MIC, where specific cellular responses can be distinguished from general toxicity.^25^ Accordingly, compounds with higher MIC values, including isoniazid (MIC > 500 μg/mL), were excluded from this analysis. Thus, the number of plots presented per species reflects the subset of compounds meeting this threshold rather than inconsistencies in data acquisition.

A limitation of this dataset is the absence of an untreated control population during flow cytometric acquisition. Nevertheless, a 50% ethanol-treated sample was included as a positive control and consistently produced a well-defined non-viable cell population, confirming staining efficiency, instrument performance, and gating reliability. The flow cytometry data are interpreted within a comparative framework, with treatment-induced shifts in population distributions evaluated relative to one another rather than to an untreated baseline. Quantitative distribution of cell populations is provided in Table S4.

*Candida auris* (Figure 3) treated with 50% ethanol (positive control) showed extensive membrane disruption, with 85.6% of cells PI^+^. Among the derivatives, Compound VI (15.6 μg/mL, 97.2%), Compound II (15.6 μg/mL, 96.1%), and Compound V (15.6 μg/mL, 93.8%) induced the highest proportions of PI^+^ cells, indicating pronounced loss of membrane integrity. Compound VII (31.2 μg/mL, 87.7%) also exhibited strong activity, comparable to that of ethanol. Moderate effects were observed for Compound III (125 μg/mL, 75.3%) and Compound VIII (31.2 μg/mL, 64.3%), while Compound I (31.2 μg/mL, 59.2%) showed the lowest activity. In contrast, amphotericin B (1.25 μg/mL) resulted in 57.2% PI^+^ cells, suggesting reduced membrane damage by amphotericin B as compared to the derivatives under these conditions.

**Figure 3:**
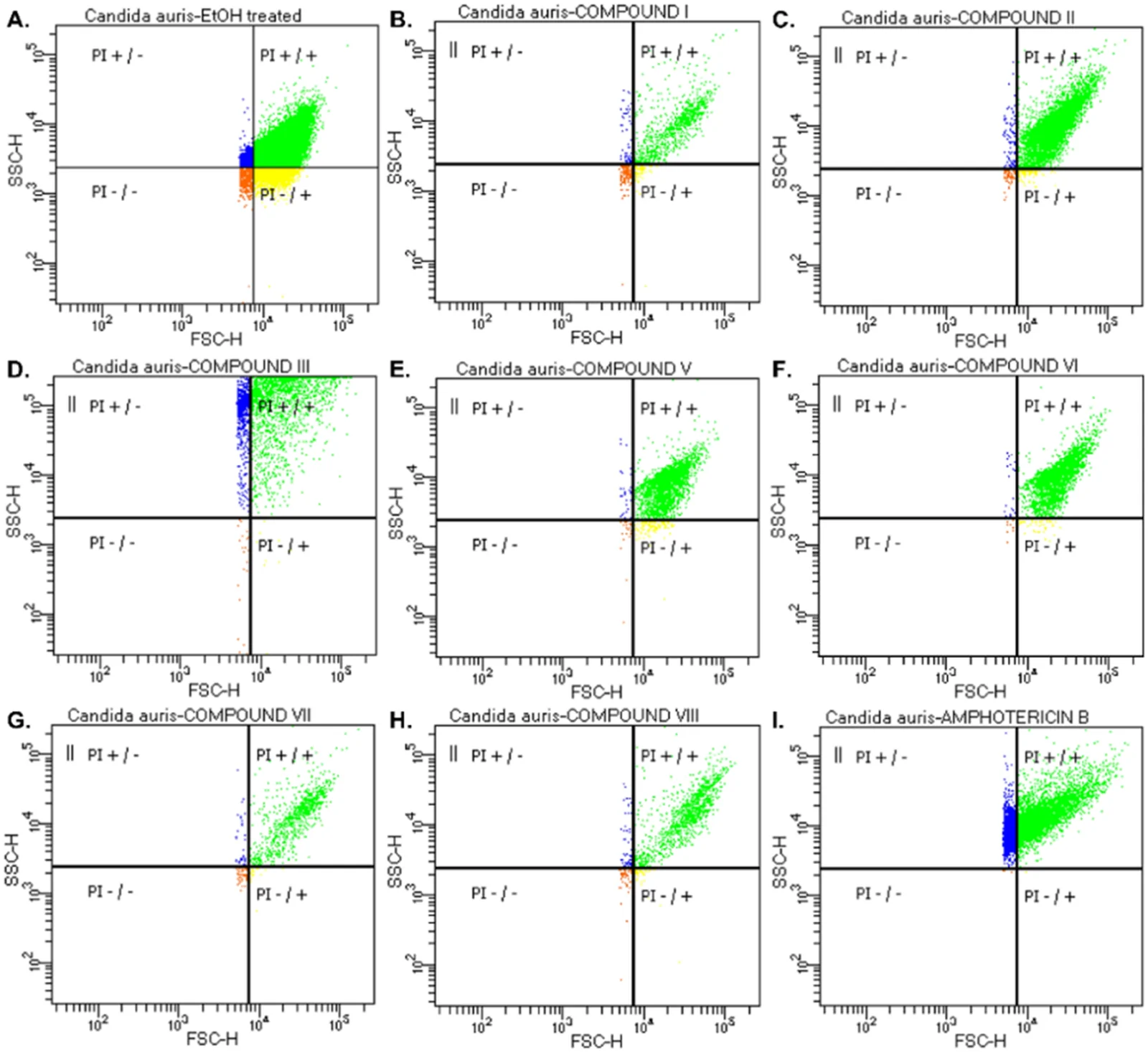
Representative FSC-H vs SSC-H scatter plots of *Candida auris* cells treated with (A) 50% ethanol (positive control), (B) Compound I (31.2 μg/mL), (C) Compound II (15.6 μg/mL), (D) Compound III (125 μg/mL), (E) Compound V (15.6 μg/mL), (F) Compound VI (15.6 μg/mL), (G) Compound VII (31.2 μg/mL), (H) Compound VIII (31.2 μg/mL) and (I) Amphotericin B (1.25 μg/mL) at their respective MIC concentrations after 24 h treatment. Data are representative of three independent experiments (*n* = 3) (BD FACSDiva software Version 8.0.1).

*Candida glabrata* (Figure 4) exposed to 50% ethanol showed near-complete membrane disruption, with 98.2% PI^+^ cells. Compound II at 62.5 μg/mL induced a similarly high proportion of PI^+^ cells (94.6%), confirming strong antifungal activity. In contrast, Compound I at 31.2 μg/mL produced a heterogeneous profile, with 47.0% PI^+^ cells and 45.5% partially compromised (PI^+^/PI^‑^), indicating mixed membrane damage. Amphotericin B at 1.25 μg/mL resulted in 60.0% PI^+^ cells, reflecting moderate disruption relative to Compound II. Compound III at 125 μg/mL yielded 76.1% PI^+^ cells, with 23.0% partially compromised.

**Figure 4:**
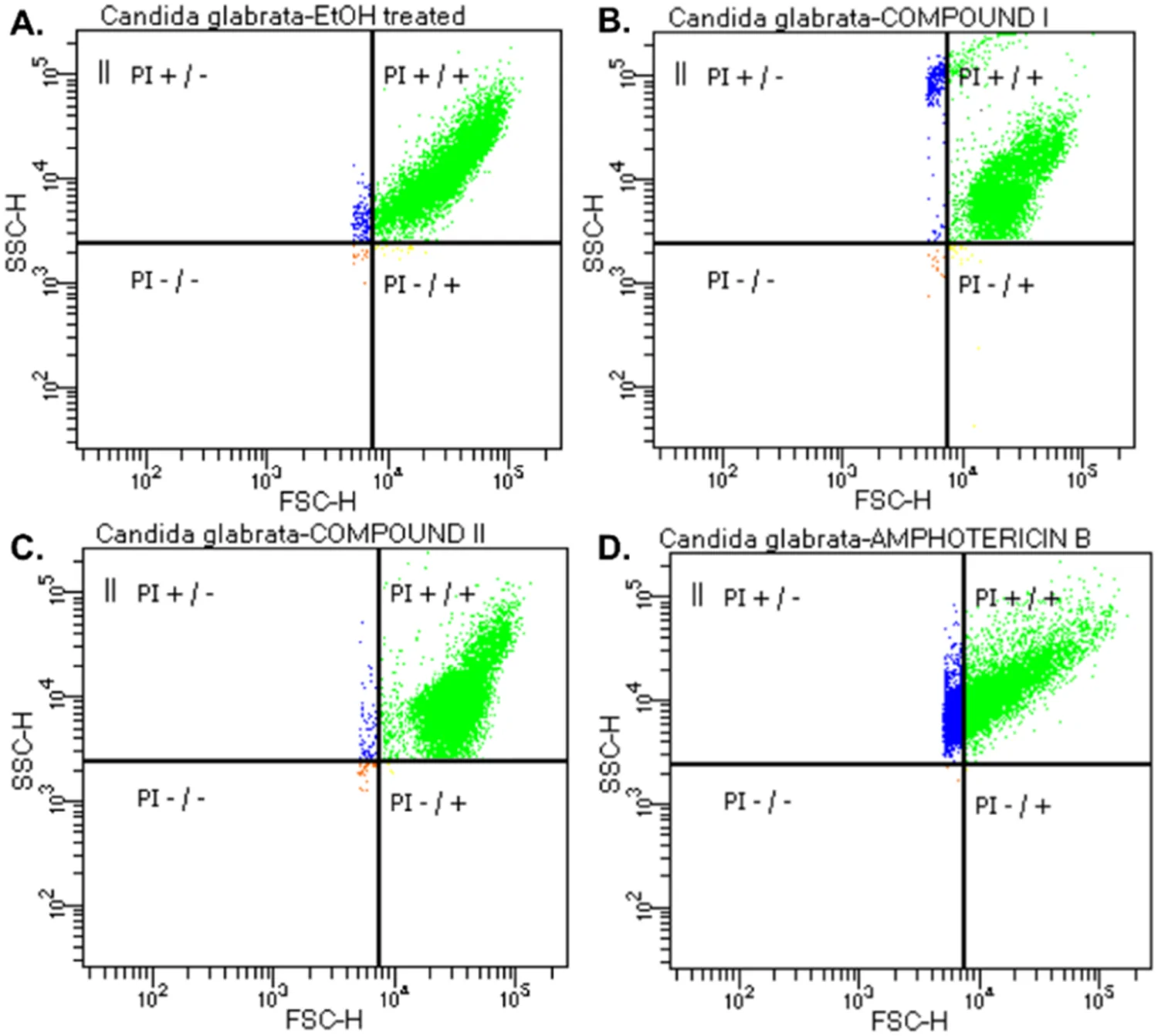
Representative FSC-H vs SSC-H scatter plots of *Candida glabrata* cells treated with (A) 50% ethanol (positive control), (B) Compound I (125 μg/mL), (C) Compound II (62.5 μg/mL), (D) Amphotericin B (1.25 μg/mL) at their respective MIC concentrations after 24 h treatment. Data are representative of three independent experiments (*n* = 3) (BD FACSDiva software Version 8.0.1).

*Candida parapsilosis* (Figure 5) treated with 50% ethanol showed extensive membrane damage, with 98.5% PI^+^ cells. All tested derivatives induced high PI^+^ populations, with Compound V (62.5 μg/mL, 94.6%), Compound VII (62.5 μg/mL, 94.0%), Compound II (31.2 μg/mL, 93.1%), Compound VIII (62.5 μg/mL, 93.0%), and Compound VI (62.5 μg/mL, 91.6%) demonstrating strong activity. In contrast, amphotericin B (62.5 μg/mL) yielded 57.9% PI^+^ cells, indicating comparatively reduced membrane disruption under these conditions.

**Figure 5:**
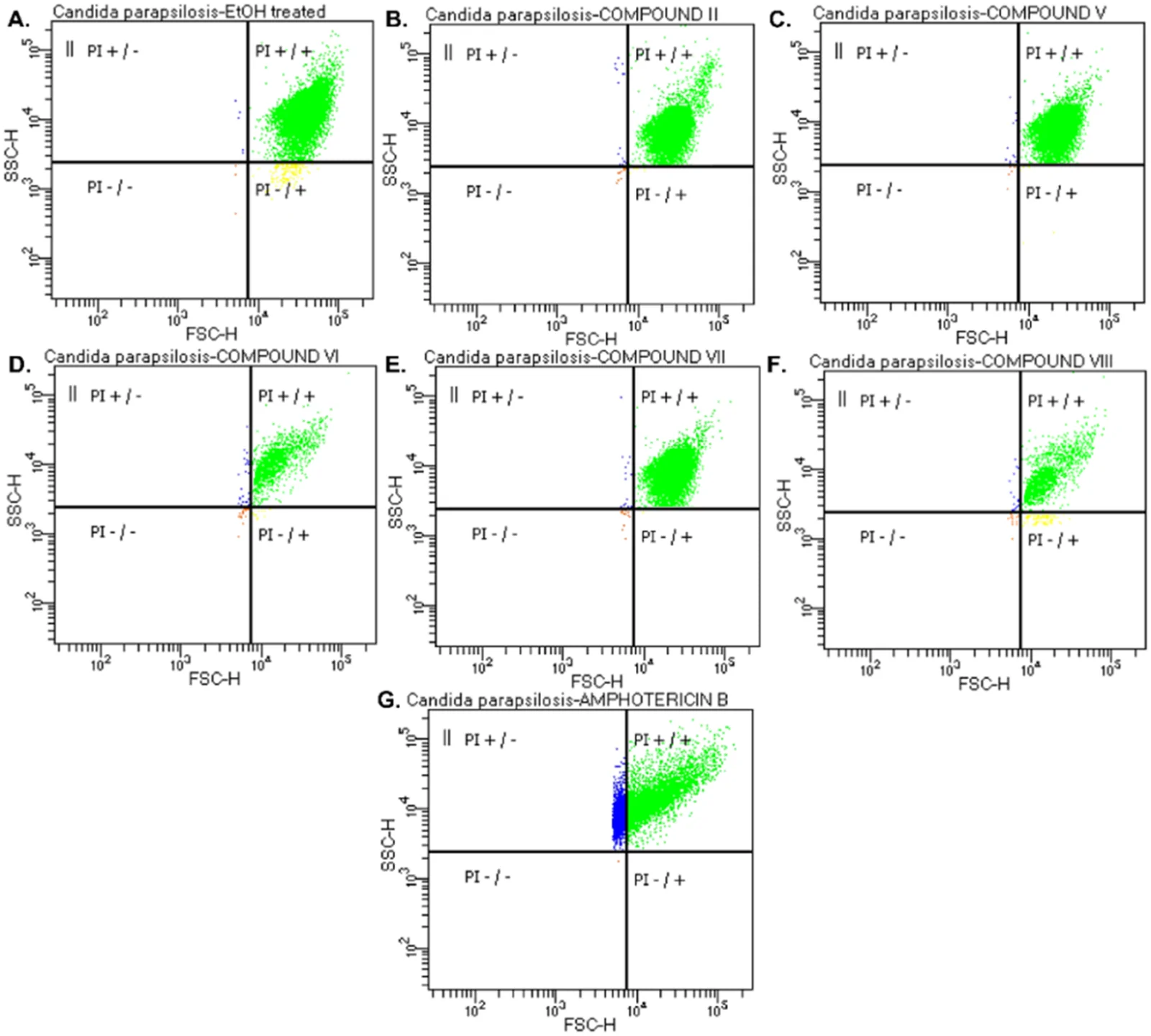
Representative FSC-H vs SSC-H scatter plots of *Candida parapsilosis* cells treated with (A) 50% ethanol (positive control), (B) Compound II (31.2 μg/mL), (C) Compound V (62.5 μg/mL), (D) Compound VI (62.5 μg/mL), (E) Compound VII (62.5 μg/mL), (F) Compound VIII (62.5 μg/mL) and (G) Amphotericin B (1.25 μg/mL) at their respective MIC concentrations after 24 h treatment. Data are representative of three independent experiments (*n* = 3) (BD FACSDiva software Version 8.0.1).

Across all three *Candida* species, several isoniazid derivatives—particularly Compounds II, V, VI, and VII—induced substantial loss of membrane integrity, as evidenced by high PI uptake. In many cases, these compounds exhibited greater membrane-disrupting effects than amphotericin B, suggesting a potent antifungal mechanism associated with cellular damage. The distribution of PI-positive populations further indicates that the compounds induce heterogeneous cell damage, ranging from partial membrane compromise to extensive loss of viability.

### Cytotoxicity Assay

To evaluate the safety profile of the compounds, cytotoxicity was assessed in mammalian cell lines (Vero) using the MTT assay. The MTT assay relies on the reduction of yellow tetrazolium salt (MTT) into purple formazan crystals by metabolically active cells. The colour change in the plates acts as a direct indicator of cell viability.^26^ The lethal concentration 50 (LC_50_) in an MTT assay is the concentration of a substance that reduces cell viability by 50% relative to untreated controls. It provides a quantitative measure of cytotoxicity, aiding in assessing the potency of a drug or compound. Cytotoxicity was evaluated using Vero (African green monkey kidney epithelial) cell lines to provide a complementary assessment of host toxicity. Vero cells are widely used in antimicrobial studies due to their stability and reproducibility and are particularly relevant for assessing renal-associated toxicity, as the kidneys are key target organs in systemic Candida infections and major sites of drug excretion.

Cytotoxicity was evaluated using the MTT assay, and LC_50_ values are reported to reflect the relative toxicity of the modified isoniazid derivatives and control compounds. Data are presented as mean ± standard deviation (SD) from three independent experiments (*n* = 3). The LC_50_ value represents the concentration of the compound required to cause 50% cell death, so lower LC_50_ values indicate greater cytotoxic potency (Table 4 and Figure 6). Compound II exhibited the highest cytotoxic potency with the lowest LC_50_ value (86.85 ± 0.2 μg/mL), suggesting strong cell-killing activity. Compound I followed with an LC_50_ value of 184.82 ± 0.2 μg/mL, indicating notable cytotoxicity but less potent than compound II. Compounds III and VII have relatively higher LC_50_ values (300.27 ± 0.2 μg/mL and 260.42 ± 0.2 μg/mL, respectively), indicating lower cytotoxicity. The addition of salicylic acid appears to influence the potency of derivatives, as seen in comparisons^27^ such as compound II, which contains salicylic acid, vs compounds I, V, and VI, which do not. Amphotericin B (AMB) shows moderate cytotoxicity with an LC_50_ of 243.98 ± 0.05 μg/mL, demonstrating its established antifungal activity but limited cytotoxic selectivity.

**Figure 6:**
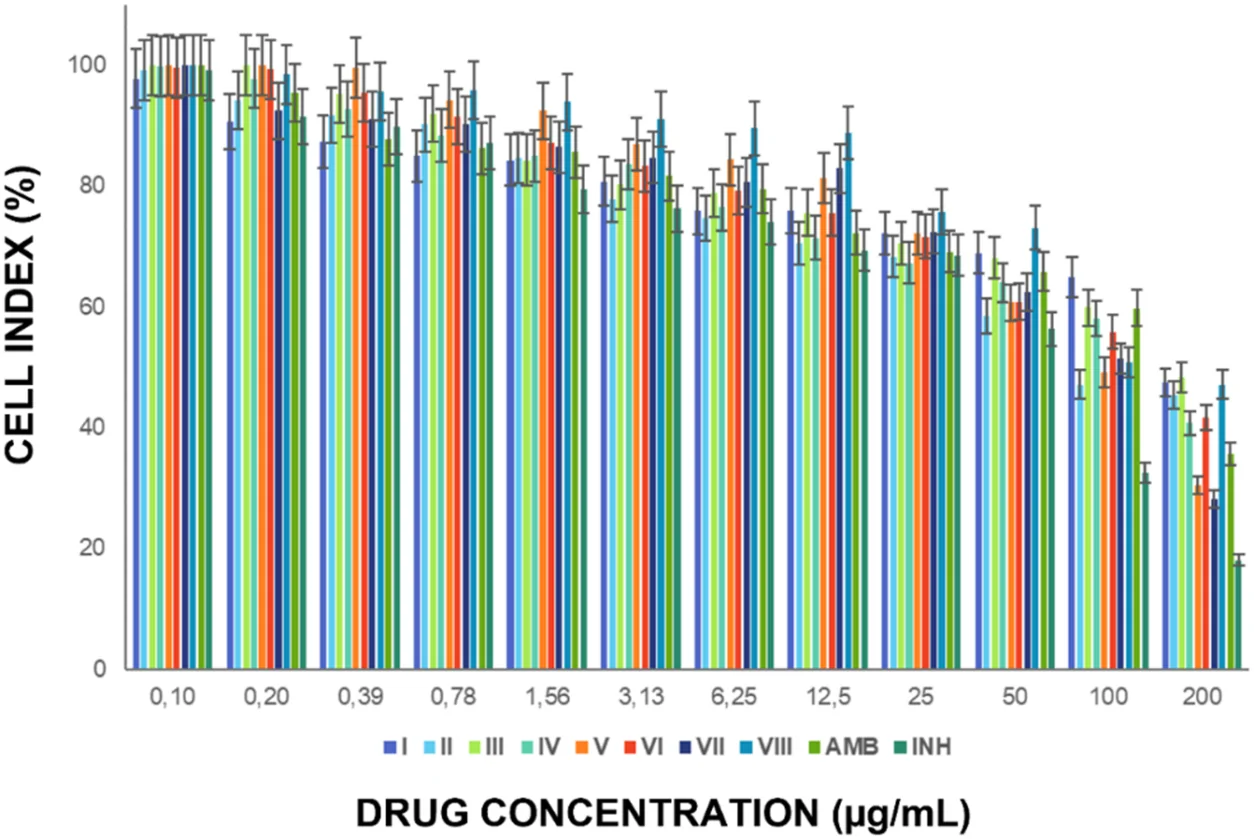
Dose-dependent cytotoxic effects of modified isoniazid derivatives, Isoniazid and Amphotericin B, on Vero cells, as measured by cell index percentage. Bar graphs illustrate real-time cell index responses across increasing drug concentrations for paired isoniazid derivatives and Amphotericin B (AMB) (GraphPad Prism Version 5.03).

**Table 4:** MTT Assay LC_50_ Values Obtained Using Modified Isoniazid Derivatives against Vero Cells^a^,^b^

Compounds	LC_50_ value (μg/mL)	LC_50_ value (μM)
Compound I	184.82 ± 0.02	554.5 ± 0.1
Compound II	86.85 ± 0.02	184.2 ± 0.1
Compound III	300.27 ± 0.02	1 091.0 ± 0.1
Compound IV	205.87 ± 0.03	453.2 ± 0.1
Compound V	128.70 ± 0.01	726.5 ± 0.1
Compound VI	145.58 ± 0.03	461.7 ± 0.1
Compound VII	260.42 ± 0.02	1 361.8 ± 0.1
Compound VIII	194.06 ± 0.04	589.3 ± 0.1
Isoniazid (INH)	87.78 ± 0.07	640.0 ± 0.5
Amphotericin B	243.98 ± 0.05	264.0 ± 0.1

a The concentrations obtained were measured in μg/mL and converted to μM. Values are expressed as mean ± SD (*n* = 3).

b *p* ≤ 0.01.

### Real-Time Cell Analysis Using xCELLigence

To complement end-point cytotoxicity data, real-time cellular responses to compound exposure were monitored using xCELLigence RTCA system. This platform enables continuous assessment of cell viability, adhesion, proliferation and morphological changes through impedance-based measurements.^28^ In this study, modified isoniazid derivatives were evaluated at their respective LC_50_ concentrations, as determined by the MTT assay, to assess dynamic cellular responses under defined cytotoxic conditions (Figure 7).

**Figure 7:**
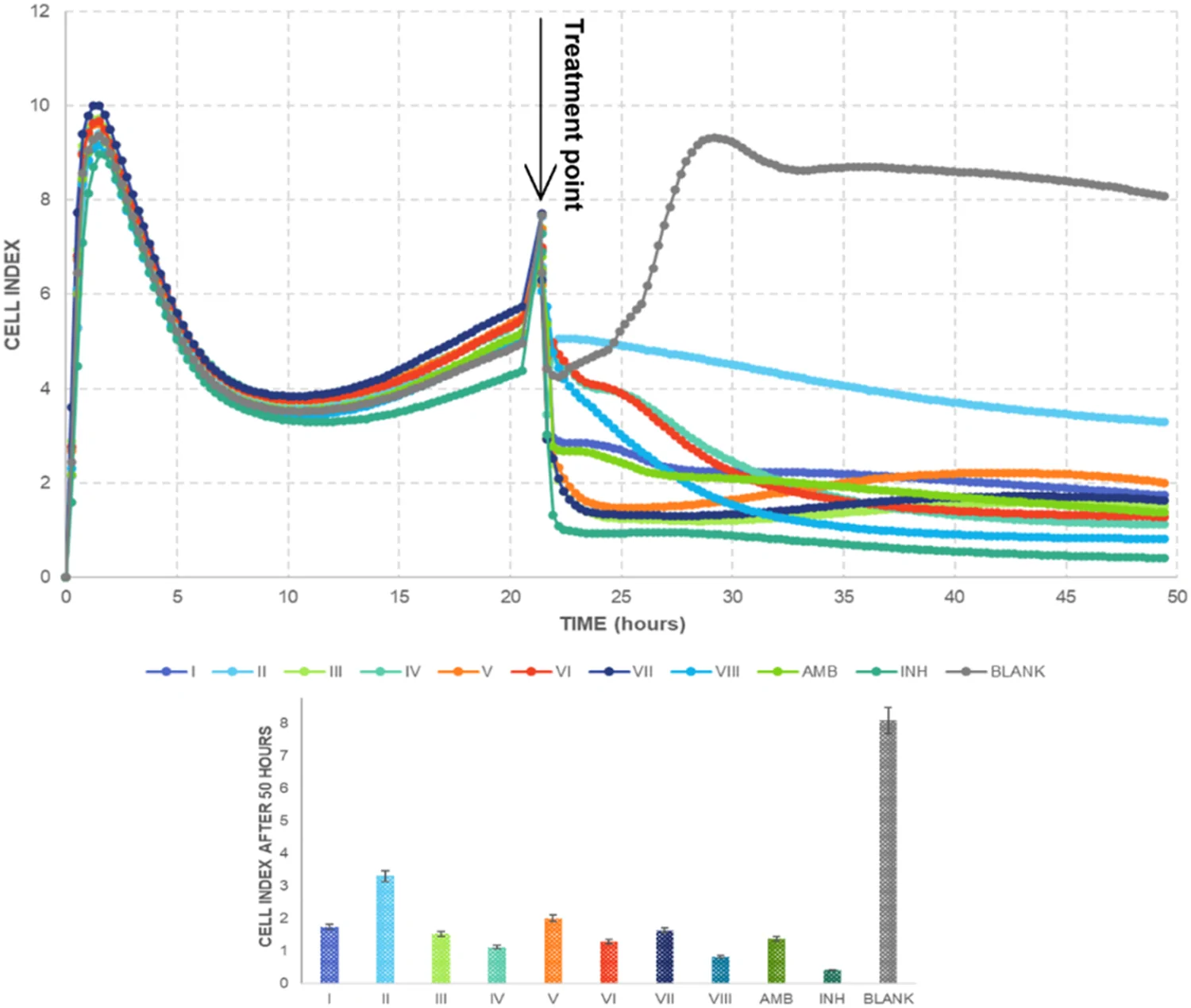
Real-time cell analysis (RTCA) of Vero cell viability following 48 h exposure to isoniazid derivatives. Cell index profiles (top) illustrate dynamic changes in cell viability over time, with compound treatment applied at the indicated time point (∼22 h). A corresponding bar graph (bottom) summarizes the cell index values at 48 h post-treatment. Compounds were tested at their respective LC_50_ concentrations: Compound I (184.82 μg/mL), Compound II (86.85 μg/mL), Compound III (300.27 μg/mL), Compound IV (205.87 μg/mL), Compound V (128.70 μg/mL), Compound VI (145.58 μg/mL), Compound VII (260.42 μg/mL), and Compound VIII (194.06 μg/mL). Isoniazid (INH, 87.78 μg/mL) and amphotericin B (AMB, 243.98 μg/mL) were included as reference controls. The untreated cells (blank) exhibited sustained growth, while treated cells showed varying degrees of cytotoxicity as reflected by reductions in cell index (xCELLigence Software Version 2.0.0.1301).

Cells were monitored over 50 h, with treatment administered at approximately 21 h post-seeding (Figure 7). Prior to treatment, all conditions exhibited comparable growth profiles, characterized by an initial adhesion phase followed by proliferation, reaching cell index values of approximately 5.0–6.0, indicating consistent cell attachment and growth.

Following treatment, a rapid decline in cell index was observed across all treated conditions, reflecting disruption of cell viability and/or adhesion. The untreated control (blank) continued to proliferate, reaching the highest cell index of approximately 8.0 and remaining stable throughout the experiment. Amphotericin B (AMB; 243.98 ± 0.05 μg/mL) induced a pronounced reduction in cell index, consistent with its known cytotoxic effects. Similarly, isoniazid (INH; 87.78 ± 0.07 μg/mL) demonstrated strong cytotoxicity, resulting in a marked and sustained decline in cell index.

Among the derivatives, Compound II (86.85 ± 0.02 μg/mL) exhibited a pronounced decrease in cell index, consistent with its relatively low LC_50_ value. In contrast, Compound III (300.27 ± 0.02 μg/mL) and Compound VII (260.42 ± 0.02 μg/mL), which possess higher LC_50_ values, showed comparatively less severe declines, suggesting reduced cytotoxicity. Compounds IV (205.87 ± 0.03 μg/mL) and VIII (194.06 ± 0.04 μg/mL) demonstrated sustained reductions in cell index, indicative of notable cytotoxic effects. Meanwhile, Compounds I (184.82 ± 0.02 μg/mL), V (128.70 ± 0.01 μg/mL), and VI (145.58 ± 0.03 μg/mL) displayed intermediate responses, reflecting moderate effects on cell viability and proliferation.

End-point analysis at 50 h supported these observations, with untreated cells maintaining the highest cell index, while treated samples exhibited reduced values in a compound-dependent manner. Overall, these findings demonstrate that the cytotoxic effects observed in RTCA broadly align with LC_50_ values obtained from MTT assays, while also revealing dynamic cellular responses that are not captured by end-point measurements alone.

### Selectivity Index (SI) Analysis and Therapeutic Potential

The selectivity index (SI), defined as the ratio of LC_50_ (mammalian cytotoxicity) to MIC (antifungal activity), was calculated to evaluate the therapeutic potential of the isoniazid derivatives across *Candida auris*, *Candida parapsilosis*, and *Candida glabrata* (Table 5). This parameter provides an indication of the balance between antifungal efficacy and host cell toxicity.

**Table 5:** Selectivity Index (SI) Values of Isoniazid Derivatives (Compounds I–VIII), Calculated as the Ratio of Cytotoxicity (LC_50_, Vero Cells) to Antifungal Activity (MIC) against *Candida auris*, *Candida parapsilosis*, and *Candida glabrata* ^a^

Compound	*Candida auris*	*Candida parapsilosis*	*Candida glabrata*
Compound I	5.92	0.74	5.92
Compound II	5.57	2.78	1.39
Compound III	2.40	1.20	2.40
Compound IV	<0.82	0.82	0.82
Compound V	8.25	2.06	0.51
Compound VI	9.33	2.33	0.58
Compound VII	8.35	4.17	1.04
Compound VIII	6.22	3.10	0.78
Isoniazid (INH)	<0.18	<0.18	<0.18
Amphotericin B	195.18	195.18	195.18

a Higher SI values indicate greater selectivity toward fungal cells relative to mammalian cells.

Against *Candida auris*, several derivatives exhibited moderate selectivity, with Compounds V (SI = 8.25), VI (SI = 9.33), and VII (SI = 8.35) demonstrating the most favorable profiles. These values suggest a reasonable balance between antifungal efficacy and cytotoxicity, highlighting these compounds as promising candidates for further investigation. In contrast, Compound IV (SI < 0.82) and isoniazid (SI < 0.18) displayed poor selectivity, indicating limited therapeutic potential. For *Candida parapsilosis*, overall selectivity was reduced across the series, with Compound VII (SI = 4.17) and Compound VIII (SI = 3.10) showing comparatively improved profiles. However, most compounds exhibited SI values below 3, suggesting modest antifungal selectivity against this species. A similar trend was observed against *Candida glabrata*, where selectivity was generally low. Only Compound I (SI = 5.92) and Compound III (SI = 2.40) demonstrated moderate activity, while the majority of derivatives showed SI values below 2, reflecting limited specificity. The reference drug Amphotericin B exhibited exceptionally high SI values (∼195 across all species), underscoring its superior therapeutic profile. However, several synthesised derivatives demonstrated moderate selectivity specifically against *C. auris*, suggesting potential for targeted antifungal development.

## Discussion

Many fungal species readily acquire antimicrobial resistance through genomic alterations ranging from point mutations and chromosomal rearrangements to the acquisition of pre-existing genetic elements and horizontal gene transfer.^29^ *Candida* species are responsible for a substantial proportion of opportunistic fungal infections, many of which have become increasingly difficult to treat due to rising resistance to conventional antifungal agents.^30^ This escalating resistance crisis underscores the urgent need for novel antifungal strategies, either as standalone therapies or in synergistic combinations, to effectively combat multidrug-resistant fungal pathogens. Drug repurposing has emerged as a promising and cost-effective approach to antifungal discovery, leveraging existing pharmacological scaffolds for new therapeutic applications.^29^ In this study, structurally modified isoniazid derivatives demonstrated significant antifungal activity against clinically relevant non-*Candida albicans* *Candida* (NCAC) species, *Candida auris*, *Candida glabrata*, and *Candida parapsilosis* specifically.

The present study demonstrates that both structural modification of the isoniazid (INH) scaffold and subsequent co-crystallization with salicylic co-formers synergistically modulate supramolecular architecture and physicochemical properties. Across Compounds I–VIII, systematic variation of substituents and co-formers resulted in pronounced differences in hydrogen bonding patterns, crystal packing, and overall lattice organization. A key observation is that co-crystallization significantly expands the hydrogen-bonding landscape of the parent INH derivatives.^31,32^ The introduction of co-formers increases the diversity of hydrogen-bond donors and acceptors, thereby enabling the formation of robust supramolecular synthons and improving lattice stabilization. In particular, co-crystals incorporating salicylic acid, such as Compounds II, IV, and VIII, consistently favor strong and directional O–H···N interactions. Such interactions with the pyridine nitrogen reinforce the well-established acid–pyridine heterosynthon. These interactions contribute to enhanced polarity and are likely to promote improved solubility and intermolecular cohesion.

Structural modification of the INH derivatives themselves further influences these interactions. Derivatives bearing hydroxyl-substituted aromatic rings display increased hydrogen bonding density due to the presence of additional O–H donor groups. This facilitates the formation of extended hydrogen-bonded networks, often resulting in two-dimensional layered structures or three-dimensional frameworks. Such architectures are indicative of efficient intermolecular connectivity and enhanced lattice stability. The incorporation of bulky substituents, such as alkyl or isopropyl groups, introduces steric constraints that disrupt optimal packing. These derivatives tend to exhibit reduced planarity and diminished π–π stacking interactions, leading to more discrete and less densely packed assemblies. In these systems, stabilization is primarily achieved through localized hydrogen bonding rather than extended supramolecular networks.

Modification of the hydrazide functionality also plays a critical role in determining synthon preference and intermolecular interactions. Compounds retaining the −CONHNH_2_ group exhibit greater hydrogen bond donor capacity, facilitating the formation of multiple stabilizing interactions. In contrast, conversion to hydrazone derivatives (–C=N–) alters the donor–acceptor balance and introduces increased conjugation, which can enhance planarity but may reduce hydrogen bond donor availability. This shift results in variations in both interaction motifs and overall packing efficiency. Taken together, these findings highlight the importance of balancing hydrogen bonding, steric effects, and π-interactions in the design of co-crystalline systems. The ability to fine-tune these parameters through targeted structural modification and co-former selection underscores the versatility of INH derivatives as platforms for crystal engineering.The structural evolution from isoniazid to Compounds I–VIII was evaluated using both ^1^H and ^13^C NMR spectroscopy. The observed resonances support successful derivatisation and hydrazone formation. Across all derivatives, the pyridine ring of the isoniazid scaffold remained clearly identifiable, with characteristic downfield ^1^H NMR resonances consistently observed between δ 8.5–8.9 ppm for the H-2/H-6 protons and δ 7.6–8.0 ppm for the H-3/H-5 protons. These deshielded resonances arise from the electron-withdrawing effect of the pyridine nitrogen. These were retained throughout the series, confirming preservation of the pyridine core following modification.^27,33,34^

The parent isoniazid spectrum displayed the expected pyridine proton pattern together with a hydrazide carbonyl resonance at δ 166.62 ppm in the ^13^C NMR spectrum. Pyridine carbons adjacent to the ring nitrogen appeared at δ 149 ppm, while pyridine CH carbons resonated near δ 121 ppm, consistent with reported spectroscopic data for isoniazid and related pyridine hydrazides.^27,35^

Formation of the hydrazone linkage in Compounds I–VIII was supported by the appearance of additional aromatic and/or aliphatic resonances relative to the parent compound. The observed downfield imine-associated resonances and expanded aromatic regions are characteristic of conjugated hydrazone systems reported in related Schiff base derivatives.^36,37^ In Compounds I–IV, aromatic resonances corresponding to *p*-hydroxyphenyl, *p*-hydroxybenzoate, salicylate, and aminophenyl substituents were observed predominantly between δ 6.6–7.9 ppm in the ^1^H NMR spectra, reflecting the increased aromatic complexity of the modified frameworks. Extensive signal overlap was observed for several derivatives, particularly Compounds II, IV, VI, and VIII, due to the presence of multiple substituted aromatic environments and conjugated systems.

The ^13^C NMR spectra further supported the incorporation of these aromatic substituents. Phenolic carbons bonded to oxygen typically resonate in the δ 155–165 ppm region due to strong deshielding effects, in agreement with the assignments observed for the hydroxyphenyl- and salicylate-containing derivatives.^38^ Phenolic carbons bonded to oxygen were consistently observed in the region δ 159–160 ppm, as seen for Compounds I, VI, and VIII, confirming the presence of salicylate or hydroxyphenyl functionalities. Additional aromatic CH and quaternary carbons appeared between δ 115–145 ppm, consistent with substituted phenyl and salicylate ring systems.

Compounds V and VI, containing dimethyl-substituted hydrazone fragments, exhibited distinct methyl proton singlets at δ 2.2–1.8 ppm in the ^1^H NMR spectra, confirming incorporation of the C=N–C­(CH_3_)_2_ moiety. Corresponding methyl carbon resonances within the expected aliphatic region further supported successful condensation reactions and hydrazone formation.^36,39^ Corresponding methyl carbon resonances were observed at δ 17–24 ppm in the ^13^C NMR spectra. In both compounds, imine carbon resonances were identified near δ 162–166 ppm, while hydrazide carbonyl carbons resonated between δ 163–169 ppm, supporting successful condensation of the hydrazide functionality.

For Compounds VI and VIII, salicylate-derived carbonyl resonances appeared near δ 175 ppm, together with phenolic carbon signals at δ 159 ppm. Aromatic proton splitting patterns observed in the ^1^H NMR spectra, including doublets of doublets and doublets of doublets of doublets, were consistent with ortho- and meta-coupled aromatic systems characteristic of substituted salicylate rings. Similar splitting patterns and chemical shift distributions have previously been reported for salicylaldehyde-derived hydrazones and related aromatic Schiff bases.^37,40^

In Compound VII, the ^1^H and ^13^C NMR spectra closely resembled those of isoniazid, and the expected alkyl resonances associated with the proposed hydrazone substituent were not clearly resolved. The absence of definitive alkyl carbon signals below δ 50 ppm limited structural confirmation by NMR alone; however, complementary SC-XRD analysis provided definitive structural validation. Exchangeable NH, NH_2_, OH, and COOH protons were generally absent or weak in the ^1^H NMR spectra due to rapid deuterium exchange in D_2_O. Broad resonances observed near δ 4.7–4.9 ppm were attributed to residual HOD, while low-intensity peaks corresponding to residual solvents or trace impurities were occasionally detected in several spectra.

The combined ^1^H and ^13^C NMR data are consistent with the proposed structures of Compounds I–VIII and provide strong spectroscopic evidence for successful hydrazone derivatisation of isoniazid. The NMR findings are further supported by FTIR, Raman spectroscopy, and single-crystal X-ray diffraction analyses.

The antioxidant profiles of the isoniazid derivatives appear to be governed primarily by their functional group composition and electronic characteristics. Structural diversification of the parent isoniazid scaffold was achieved through substitution at the amino (−NH_2_) group. Introducing a range of moieties, including amino radicals, hydroxyl groups, phenolic systems, and carboxylic acid functionalities. Several derivatives further incorporated salicylic acid motifs through cocrystal formation. Thus, increasing chemical and electronic diversity. Phenolic structures, defined by aromatic rings bearing one or more hydroxyl substituents, are well established as effective antioxidants.^41,42^ Phenolic acids, which combine hydroxyl and carboxylic acid functionalities, are particularly efficient radical scavengers. This property is owed to the enhancement of the resulting phenoxyl radicals.^43^ Consistent with these principles, derivatives enriched in phenolic functionalities displayed superior antioxidant activity.^44^ Compound IV represents a notable example, as it contains multiple phenolic features, including a phenolic aromatic ring, a phenolic acid moiety, and an aniline-linked phenolic group. The coexistence of these functionalities likely promotes efficient hydrogen atom donation and of radical species, accounting for its elevated scavenging activity.^43^ The presence of salicylic acid-derived motifs may further enhance antioxidant behaviour through additional unknown resonance pathways.

Compound I also exhibited measurable antioxidant activity, which can be attributed to the presence of hydroxyl (−OH) groups capable of hydrogen atom transfer to neutralize free radicals.^19^ However, despite bearing multiple hydroxyl substituents, its activity was remarkably lower than that of compounds IV and VII. This discrepancy may arise from steric constraints or suboptimal spatial orientation of the hydroxyl groups. These are factors known to modulate antioxidant efficiency by limiting radical accessibility or resonance stabilization.^43,45^ Compound VII demonstrated the strongest radical scavenging activity in the series, with an effective concentration approaching that of ascorbic acid. This enhanced performance may be partly attributed to the presence of a butan-2-ylidene substituent.^20,46^ A molecule of lipophilicity and a facilitator of lipid-associated radical species interactions. Increased lipophilicity has been associated with improved membrane integration and more efficient scavenging of lipid-derived radicals.^47^ Additionally, this substituent may modulate electron density distribution within the molecular framework. Favoring hydrogen atom donation or single-electron transfer mechanisms.

The antifungal activity of the modified isoniazid derivatives against non-*Candida albicans* *Candida* (NCAC) species was assessed using minimum inhibitory concentration (MIC) assays. Several derivatives demonstrated notable antifungal potency, with many exhibiting superior activities relative to the positive control, amphotericin B (AMB). Based on the criteria proposed by ref 48, MIC values in the range of 64 to 100 μg/mL are considered clinically relevant. Importantly, all modified isoniazid derivatives produced inhibitory concentrations within or below this threshold against at least one of the tested *Candida* species, underscoring their potential as antifungal agents. Compounds II, VI, and VII, which each incorporate a salicylic acid co-former, demonstrated activity across multiple *Candida* species. The inclusion of salicylic acid may contribute to the observed antimicrobial effects, consistent with previous reports.^49,50^ However, its specific role in enhancing activity in this system remains to be fully elucidated.

Comparative evaluation against *Candida auris*, *Candida glabrata*, and *Candida parapsilosis* revealed pronounced species-dependent differences in antifungal susceptibility. Compounds II, V, and VI exhibited the strongest activity against *C. auris*, with MIC values of 15.63 μg/mL, however substantially outperformed INH, which presented with an MIC value greater than 500 μg/mL. This marked enhanced potency indicates effective suppression of fungal growth and highlights these derivatives as promising candidates for further investigation. Against *C. parapsilosis*, compound II again demonstrated the greatest activity, exceeding that of INH (>500 μg/mL). Compounds V, VI, VII, and VIII displayed moderate activity with MICs of 125 μg/mL. In contrast, compounds I, III, and IV exhibited limited efficacy against this species. Distinct trends were observed for *C. glabrata*, where Compound I emerged as the most potent derivative, exhibiting an MIC value of 31.2 μg/mL, again surpassing INH (>500 μg/mL). Other derivatives, including compounds V, VI, VII, and VIII, showed considerably weaker activity with MIC values greater than 250 μg/mL. Highlighting pronounced strain-specific variability in antifungal responsiveness. Amphotericin B maintained consistent MIC values of 1.25 μg/mL across all tested species, serving as a reliable benchmark for comparative assessment.^51,52^

The observed variability in antifungal activity among the derivatives underscores the critical role of structural modification in modulating antifungal potency. Functional groups that enhance bioavailability, membrane permeability, or target engagement likely contribute to improved efficacy, as exemplified by compound II. This compound demonstrated strong and consistent activity across all NCAC species evaluated. Compounds V and VI, while highly effective against *C. auris* and *C. parapsilosis*, exhibited diminished activity against *C. glabrata*. These observations suggest the presence of species-specific interactions or uptake mechanisms. The reduced activity observed for compounds III and IV may be attributed to steric hindrance or limited penetration into fungal cells, which can restrict antifungal effectiveness.^48^

A key mechanistic observation from this study is the induction of programmed cell death in fungal cells following exposure to the most active isoniazid derivatives. Flow cytometric analysis using propidium iodide (PI) staining provided mechanistic insight into the antifungal effects of the synthesised isoniazid derivatives by assessing membrane integrity in *Candida* species.^53^ The significant increase in PI-positive populations across treated samples indicates that the compounds induce loss of membrane integrity, consistent with cell death. Notably, Compounds II, V, VI, and VII consistently produced the highest levels of PI uptake across *Candida auris*, *Candida glabrata*, and *Candida parapsilosis*, suggesting that these derivatives exert pronounced membrane-disruptive effects.

The observed biological activity can be rationalized in terms of the structural modifications introduced onto the isoniazid scaffold. Derivatives incorporating aromatic substituents and extended conjugation (Compounds II and IV) are likely to enhance lipophilicity, facilitating improved interaction with the fungal cell membrane. Increased lipophilicity may promote partitioning into the lipid bilayer, thereby destabilizing membrane structure and increasing permeability, which is consistent with the high PI uptake observed for Compound II.

Similarly, derivatives bearing phenolic functionalities (Compounds III and V) introduce additional hydrogen-bonding capacity and redox-active sites. These groups may contribute to oxidative stress or membrane perturbation through interactions with membrane-associated proteins and lipids.^54^ Compound V, in particular, demonstrated strong PI uptake, suggesting that the presence of hydroxyl groups enhances its ability to disrupt membrane integrity, possibly through increased polarity combined with localized hydrogen bonding.

In contrast, derivatives containing bulkier substituents or reduced hydrogen-bonding capacity (Compounds I and VIII) exhibited comparatively lower PI-positive populations. This reduced activity may be attributed to steric hindrance, which limits efficient interaction with the membrane, or to a suboptimal balance between hydrophilicity and lipophilicity, thereby reducing cellular uptake and membrane association.

The acetone- and butanone-derived hydrazone modifications (Compounds VI and VII) appear to play a critical role in enhancing antifungal activity.^46^ These smaller, more flexible substituents may allow better accommodation within the lipid bilayer, facilitating membrane disruption without excessive steric constraints. Compound VI, in particular, showed one of the highest levels of PI uptake, suggesting that such modifications optimize the balance between molecular size, flexibility, and lipophilicity.^55^

When compared to amphotericin B, a known ergosterol-binding antifungal agent, several of the synthesised derivatives induced equal or greater levels of membrane damage, as evidenced by PI staining. This suggests that, although the mechanism may differ, the overall effect on membrane integrity is comparable. Unlike amphotericin B, which forms defined membrane pores, the isoniazid derivatives may act through non-specific membrane destabilization or indirect stress-mediated pathways, potentially involving disruption of lipid organization or induction of oxidative damage.^51^ It is important to note that PI staining alone does not distinguish between apoptotic and necrotic cell death pathways. Therefore, while the data clearly demonstrate membrane disruption and loss of viability, the precise mode of cell death cannot be definitively assigned. Nonetheless, the strong correlation between PI uptake, MIC values, and structural features supports a mechanism in which membrane perturbation is a key contributor to antifungal activity. These findings highlight the importance of structural tuning of the isoniazid scaffold, where the introduction of lipophilic, phenolic, and flexible substituents enhances membrane interaction and antifungal efficacy.^55^ This structure–activity relationship provides valuable insight for the rational design of future derivatives with improved potency and selectivity.

When considered alongside the antifungal MIC data, the cytotoxicity findings provide an initial assessment of the therapeutic index for the modified isoniazid derivatives. Compounds that combine low MIC values against NCAC species with minimal effects on Vero cell viability, such as compound III, exhibit a more favorable *in vitro* therapeutic window. In contrast, compound II, despite demonstrating potent antifungal activity, displayed comparatively higher cytotoxicity toward mammalian cells, suggesting a narrower therapeutic margin at the concentrations evaluated. These observations emphasize that antifungal potency alone is insufficient to predict clinical utility and highlight the importance of balancing fungal growth inhibition with host cell compatibility during lead optimization.^56^ Derivatives exhibiting moderate antifungal activity but reduced cytotoxicity, including compounds III and IV, may therefore represent more promising scaffolds, as their safety profile could potentially be improved without substantial loss of antifungal efficacy. On the other hand, highly potent but more cytotoxic derivatives may require additional structural refinement or formulation strategies to enhance selectivity. Overall, integration of MIC and cytotoxicity data underscores the necessity of considering therapeutic index early in antifungal development. This supports prioritisation of compounds with both effective antifungal activity and acceptable mammalian cell tolerance for subsequent *in vivo* evaluation.

## Conclusion

The evaluation of modified isoniazid derivatives highlights the crucial role of structural features in determining antioxidant efficacy. Compound I, which incorporates phenolic hydroxyl (−OH) groups, demonstrates notable radical scavenging activity, supporting previous findings that hydroxyl positioning on the benzene ring directly influences antioxidant potential. While this compound has a strong hydrogen-donating ability, its lower antioxidant capacity compared to previously discussed derivatives suggests that steric hindrance may limit optimal radical interaction. Similarly, compound VII facilitates improved integration into biological membranes and enhances the efficiency of radical neutralization. Its contribution to electron density distribution further supports its hydrogen donation potential, which may explain its lower scavenging concentration, closely aligning with the activity of ascorbic acid.

The antifungal activity assessment of modified isoniazid derivatives against *Candida auris*, *Candida glabrata*, and *Candida parapsilosis* underscores the impact of structural modifications on antifungal efficacy. Several derivatives, particularly compounds II, V, and VI, exhibited superior potency compared to Amphotericin B (AMB), reinforcing their potential as promising antifungal candidates. The observed variations in minimum inhibitory concentrations (MICs) suggest species-specific interactions, where compounds that were highly effective against *Candida auris* and *Candida parapsilosis* demonstrated limited activity against *Candida glabrata*. This highlights the intricate relationship between molecular composition and fungal uptake mechanisms. Among the tested derivatives, compound II displayed broad-spectrum potency, likely benefiting from enhanced lipophilicity or electron-donating functional groups that improve bioavailability. Conversely, compounds III and IV showed weaker efficacy, potentially due to steric hindrance or poor permeability. The comparative analysis with AMB further emphasizes the therapeutic relevance of these novel derivatives, as multiple compounds exhibited significantly lower MIC values, indicating stronger antifungal activity.

While AMB is effective against fungal pathogens, concerns regarding its renal toxicity necessitate careful consideration of its broader therapeutic applications. Given the observed trends, structural modifications of isoniazid that incorporate salicylic acid appear to enhance cytotoxic potency. However, optimizing selectivity is crucial; high potency must be balanced with therapeutic viability to avoid excessive toxicity in normal cells. Future studies could explore the mechanistic pathways underlying these trends, including apoptosis markers and oxidative stress indicators, to better understand the cellular response to these derivatives. Expanding the scope to include additional cell lines may also provide broader insights into their pharmacological relevance.

## Methods and Materials

### Synthesis and Characterization of Isoniazid Derivatives

Six isoniazid derivatives were synthesised at the Infectious Disease Research Unit, University of South Africa. Compounds I–VIII were synthesised via condensation of isoniazid (INH) with the appropriate modifiers under reflux conditions, followed by slow evaporation to yield crystalline products.

For Compound I, isoniazid (0.21 g, 1.53 mmol) was reacted with 2,2-dihydroxybenzophenone in methanol in the presence of 4-hydroxybenzoic acid (0.21 g, 1.52 mmol). The reaction was conducted in a sealed vial at 90 °C for 24 h.^14^ Compound II, isoniazid (0.21 g, 1.53 mmol) and 2,2-dihydroxybenzophenone were combined with salicylic acid in acetonitrile. Nickel nitrate was used as a catalyst. The reaction was heated at 90 °C for 96 h.^15^ Compound III, isoniazid (0.22 g, 1.60 mmol), was reacted with 4-aminobenzophenone in methanol using *p*-toluenesulfonic acid as a catalyst at 110 °C for 96 h.^16^ Compound IV, isoniazid (0.21 g, 1.53 mmol) and 4-aminobenzophenone, were reacted with salicylic acid in methanol in the presence of 0.20 g of nickel nitrate. The mixture was heated at 60 °C for 24 h.^17^ Compound V, isoniazid (0.22 g, 1.60 mmol), was treated with acetone in a sealed vial at 60 °C for 24 h without additional catalyst or co-former.^18^ Compound VI, isoniazid (0.32 g, 2.33 mmol) was reacted with acetone in the presence of 0.23 g of salicylic acid at 60 °C for 24 h.^18^ Compound VII, isoniazid (0.22 g, 1.60 mmol) was treated with 2-butanone under sealed conditions at 60 °C for 24 h.^19^ Compound VIII, isoniazid (0.22 g, 1.60 mmol) was reacted with 2-butanone in the presence of 0.22 g of salicylic acid at 60 °C for 24 h.^20^

In all cases, after completion of the reaction, the mixtures were allowed to cool to room temperature and subjected to slow solvent evaporation, yielding crystalline products suitable for further characterization.

### Fourier Transform Infrared (FTIR) and Raman Spectroscopy

Fourier transform infrared (FTIR) spectra were recorded at 288.15 K using a Shimadzu IR spectrometer equipped with a QATR10 attenuated total reflectance (ATR) accessory fitted with a germanium crystal. Spectra were acquired in percentage transmittance mode over the range of 4000–700 cm^–1^, with a spectral resolution of 4 cm^–1^ and 64 accumulated scans per sample. Data acquisition and processing were performed using LabSolutions IR (version 2.26).

Raman spectra were collected using a Bruker MultiRam Fourier-transform Raman spectrometer equipped with a Nd:YAG laser (λ = 1064 nm) and a germanium diode detector. Measurements were conducted with 64 scans at a laser power of 450 mW. All spectra were recorded under ambient conditions unless otherwise stated.

### Nuclear Magnetic Resonance (NMR) Spectroscopy

Nuclear magnetic resonance (NMR) spectra were recorded using an Agilent Technologies 500 MHz spectrometer at 299 K. Samples were prepared by dissolving the crystalline compounds in deuterated water (D_2_O) containing tetramethylsilane (TMS, 1%) as an internal standard, followed by transfer into standard 5 mm NMR tubes.

^1^H NMR spectra were acquired at 500 MHz, and ^13^C NMR spectra at 125 MHz. Chemical shifts (δ) are reported in parts per million (ppm) relative to TMS. All spectra were processed using standard instrument software, and peak assignments were made based on chemical shift, multiplicity, and integration where applicable.

### Antioxidant Activity Assay

The DPPH assay was performed following^57^ with modifications. Isoniazid derivatives (10 μL) were diluted in methanol (100 μL) to obtain various serially diluted concentrations. A 0.04 mg/mL DPPH solution (Sigma-Aldrich, Germany) was added to a microtiter plate under dim light. Samples were incubated at room temperature, in the dark, for 1 h. Absorbance was measured at 517 nm using a UV-spectrophotometer (Thermo Scientific, Varioskan Flash, Finland). Ascorbic acid (5 mg/mL, Sigma-Aldrich, Japan) was the positive control, while distilled water was the negative control. Assays were conducted in triplicate, and the radical scavenging activity (RSA) was calculated as

$$R\mathrm{SA}=\left(1-\left(A_{E}/A_{D}\right)\right)\times 100$$

Where *A*_E_ is the absorbance of the derivative or ascorbic acid mixture minus its inherent colour, and *A*_D_ is the absorbance of DPPH alone.

Dose-response curves were constructed by plotting percentage inhibition against the logarithm of compound concentration. The half-maximal inhibitory concentration (IC_50_) values were determined by nonlinear regression analysis using a four-parameter logistic (4PL) model. The goodness of fit was evaluated using the coefficient of determination (*R* ^2^), with values greater than 0.9 considered indicative of an acceptable fit.

### Candida Cultures

*Candida auris*, *Candida glabrata*, and *Candida parapsilosis* were obtained as lyophilized pellets from Microbiologics KWIK STIK devices. Each strain was subcultured onto Sabouraud Dextrose Agar (SDA) and incubated at 37 °C for 24–72 h to obtain pure colonies. Well-isolated colonies were suspended in sterile 0.85% (w/v) sodium chloride solution and adjusted to a 0.5 McFarland standard, corresponding to approximately 1–5 × 10^6^ CFU/mL. Before susceptibility testing, the suspension was further diluted in broth medium to obtain the final working inoculum.

### Minimum Inhibitory Concentration

Minimum inhibitory concentration (MIC) of each modified isoniazid derivative was determined using a broth microdilution assay adapted from ref 57 and performed in alignment with CLSI M27-A4 guidelines for antifungal susceptibility testing. Briefly, compounds were prepared in Sabouraud Dextrose Broth (SDB) and dispensed into the first row of sterile 96-well microtiter plates, followed by two-fold serial dilutions across the plate. Amphotericin B (Sigma-Aldrich, Israel) was included as a positive control, alongside growth and sterility controls.

Standardized *Candida* inocula were added to each well at a volume of 100 μL to achieve a final inoculum density of approximately 0.5–2.5 × 10^3^ CFU/mL. Plates were incubated at 37 °C for 24 h. Following incubation, 0.4 mg/mL *p*-iodonitrotetrazolium chloride (INT) (Sigma-Aldrich, Austria) was added to each well, and plates were incubated for a further 24 h. Wells exhibiting red colouration indicated metabolically active fungal growth, whereas the absence of colour change indicated growth inhibition. MIC values were defined as the lowest compound concentration preventing INT reduction. All experiments were performed in triplicate across three independent assays.

### Flow Cytometry

Cell viability and membrane integrity were assessed using propidium iodide (PI) staining following a modified method of ref 58. *Candida* species were cultured on Sabouraud Dextrose Agar (SDA) and transferred into Sabouraud Dextrose Broth (SDB), followed by incubation for 24 h at 37 °C with agitation (160 rpm). Cells were harvested in the exponential growth phase, washed with sterile saline and adjusted to a 0.5 McFarland standard.

Cell suspensions were exposed to the respective compounds at their minimum inhibitory concentrations (MICs) and incubated for 24 h. Cells treated with 50% ethanol served as a positive control for membrane damage. Following incubation, cells were collected by centrifugation, washed, and resuspended in saline solution. Cells were stained with propidium iodide (PI) and incubated in the dark for 30 min. PI is a membrane-impermeable DNA intercalating dye that selectively stains cells with compromised membrane integrity, allowing discrimination between viable (PI-negative) and non-viable (PI-positive) populations.

Flow cytometric analysis was performed using a BD FACSAria III cell sorter (BD Biosciences, San Jose, CA, USA) equipped with a 488 nm argon laser and a 70 μm nozzle. PI fluorescence was detected in the red channel (FL3, λ_em_ > 600 nm). Cell populations were initially gated based on forward scatter (FSC-H) and side scatter (SSC-H) parameters to exclude debris and to assess cell size and internal complexity. Quantification of cell viability was based solely on PI fluorescence intensity. Data acquisition and analysis were performed using BD FACSDiva software (Version 8.0.1).

### Cell Maintenance

Cytotoxicity of compounds was screened on Vero Monkey Kidney cells (Separation Scientific Cellonex, passage #9, lot: 1, South Africa) (African green monkey kidney cells) was assessed using the [3-(4,5-dimethylthiazol-2-yl)-2,5-diphenyltetrazolium bromide] (MTT) tetrazolium reduction assay. Cells were cultured in Dulbecco’s Modified Eagle Media (DMEM) (Hyclone, USA) supplemented with Fetal Bovine Serum (FBS) (Hyclone, South America) and 1% Penicillin/Streptomycin (Pen/Strep) (Separations Scientific, South Africa). Cells were maintained until reaching approximately 90% confluency.

### Cytotoxicity Assay

Cytotoxicity of each isoniazid derivative was assessed using the MTT colourimetric assay, following the method described by ref 59. The experiment was performed in 96-well microtiter plates, where Vero cells were seeded in a monolayer at a density of 1.12 × 10^5^ cells/well. After attachment, the media in each well was aspirated and replaced with various concentration of isoniazid derivatives in media. These derivatives were serially diluted in a separate plate to achieve various concentrations before being transferred to the 96-well plate containing confluent cells. The plates were incubated at 37 °C in a 5% CO_2_ chamber for 48 h. Following incubation, the cells were treated with 50 μL of MTT solution (Sigma-Aldrich, Germany) and incubated for 3 h. Subsequently, 100 μL of dimethyl sulfoxide (DMSO) (Promark Chemicals, South Africa) was added to each well and incubated for 1 h to dissolve the formazan crystals formed. The absorbance was measured at 570 nm with a reference wavelength of 630 nm using a Thermo Scientific Varioskan Flash UV-spectrophotometer (Finland). Untreated cells served as the negative control, and those treated with amphotericin B (AMB) served as the positive control. Cell viability was calculated using the following formula

$$\mathrm{cell viability}\;\left(\%\right)=\left(A_{T}/A_{C}\right)\times 100$$

*A*_T_: absorbance of derivative-treated cells;

*A*_C_: absorbance of untreated cells.

### Real-Time Cell Analysis

The real-time effects of the compounds on Vero cells were assessed using the RTCA-DP system (xCELLigence, ACEA Biosciences, Roche Applied Science, USA) following modifications to the method by ref 60. Cells were cultured in flasks until reaching 80–90% confluency, then seeded into E-plates at a density of 1.1 × 10^5^ cells/mL and incubated for 24 h before treatment. Compounds were administered at their 50% lethal concentration (LC_50_), which was determined in the previously described experiment. The experiment was conducted in duplicates, where cellular response was monitored over 24 h using xCELLigence software (version 2.0.0.1301).

## Supplementary Material



## Notes

Additional results, including FTIR, Raman, and NMR spectra, spectral assignment, FTIR and Raman spectra for compounds, FTIR and Raman spectra for isoniazid, and flow cytometry data (PDF)

## Abbreviations

NCAC: non-*Candida albicans* *Candida*
INH: isonicotinic acid hydrazide or isoniazid
IC_50_: half maximal inhibitory concentration
LC_50_: half-maximal lethal concentration
MIC: minimum inhibitory concentration
