# Supplementary material for: Beyond Tuberculosis: Unlocking the Antifungal Potential of Novel Isoniazid Derivatives against Non-Candida albicans Candida Species
Source: ACS Omega. 2026 Sep 11;11(37):55020–37. doi: 10.1021/acsomega.6c00859 (PMC13613473; doi:10.1021/acsomega.6c00859)
Supplement: Supplementary file 1 [file ao6c00859_si_001.pdf]

Beyond Tuberculosis: Unlocking the Antifungal Potential of Novel Isoniazid Derivatives against Non-*Candida albicans* Candida Species

Tebogo M. L. Mokoto[1] and Itumeleng B. Setshedi[2*]

[1]Biotechnology Niche Area, College of Science, Engineering and Technology, University of South Africa, Gauteng, South Africa, [2]Department of Life and Consumer Science, College of Agriculture and Environmental Science, University of South Africa, Gauteng, South Africa.

Keywords

Isoniazid derivatives, Drug repurposing, Non-Candida albicans Candida (NCAC), Antifungal activity, Membrane integrity, Antimicrobial resistance, Cytotoxicity

SUPPLEMENTARY INFORMATION

Table S1: Selected FTIR and Raman vibrational bands (cm^{-1}) and their assignments for isoniazid and Compounds I–VIII.

Compound	FTIR (cm^{-1})	Assignment	Raman (cm^{-1})	Assignment
Parent (Isoniazid)	3300–3400	N–H stretching (hydrazide)	~1600–1620	Aromatic C=C/C=N (pyridine ring)
	1650–1700	Amide I (C=O)	1000–1200	Pyridine ring breathing
	~1550–1560	Amide II (N–H bend + C–N)		—
	1000–1200	C–N stretching	—	—
I	3300–3400	N–H stretching	~1620	C=N stretching (hydrazone)
	1650–1700	Amide I (C=O)	1000–1200	Aromatic ring breathing
	~1550–1560	Amide II	~1600	Aromatic C=C
II *(co-crystal)*	3300–3400 (broad)	O–H/N–H (H-bonded)	~1600	Aromatic ring (salicylic acid + backbone)
	1650–1700 (shifted)	C=O (amide + COOH)	~1400	COOH deformation
	~1550–1560 (modified)	Amide II	~1620	C=N (hydrazone)
III	3300–3400	N–H stretching (less broadened)	~1620	C=N (hydrazone)

	1650–1700	Amide I (C=O)	1000–1200	Pyridine/aromatic breathing
	~1550–1560	Amide II	~1600	Weak aromatic C=C
IV *(co-crystal)*	3300–3400 (broad)	O–H/N–H (H-bonded)	~1600	Aromatic ring (enhanced)
	1650–1700 (shifted)	C=O (amide + COOH)	~1400	COOH deformation
	~1550–1560 (sharp)	Amide II	~1620	C=N (hydrazone)
V	3300–3400	N–H stretching	~1450	C–H deformation (isopropyl)
	1650–1700	Amide I (C=O)	~1620	C=N (hydrazone)
	~1550–1560	Amide II	1000–1200	Aromatic ring breathing
VI *(co-crystal)*	3300–3400 (broad)	O–H/N–H (H-bonded)	~1600	Aromatic ring
	1650–1700 (shifted)	C=O (amide + COOH)	~1400	COOH deformation
	~1550–1560 (sharp)	Amide II	~1620	C=N (hydrazone)
VII *(hydrate)*	3300–3400 (broad)	O–H/N–H (hydration + N–H)	~1640	H_2O bending
	1650–1700	Amide I (C=O)	~1600	Aromatic ring
	~1550–1560	Amide II	~1620	C=N (hydrazone)
VIII *(co-crystal)*	3300–3400 (very broad)	O–H/N–H (H-bonded)	~1600	Aromatic ring
	1650–1700 (shifted)	C=O (amide + COOH)	~1400	COOH deformation

	~1550–1560 (intense)	Amide II	~1620	C=N (hydrazone)

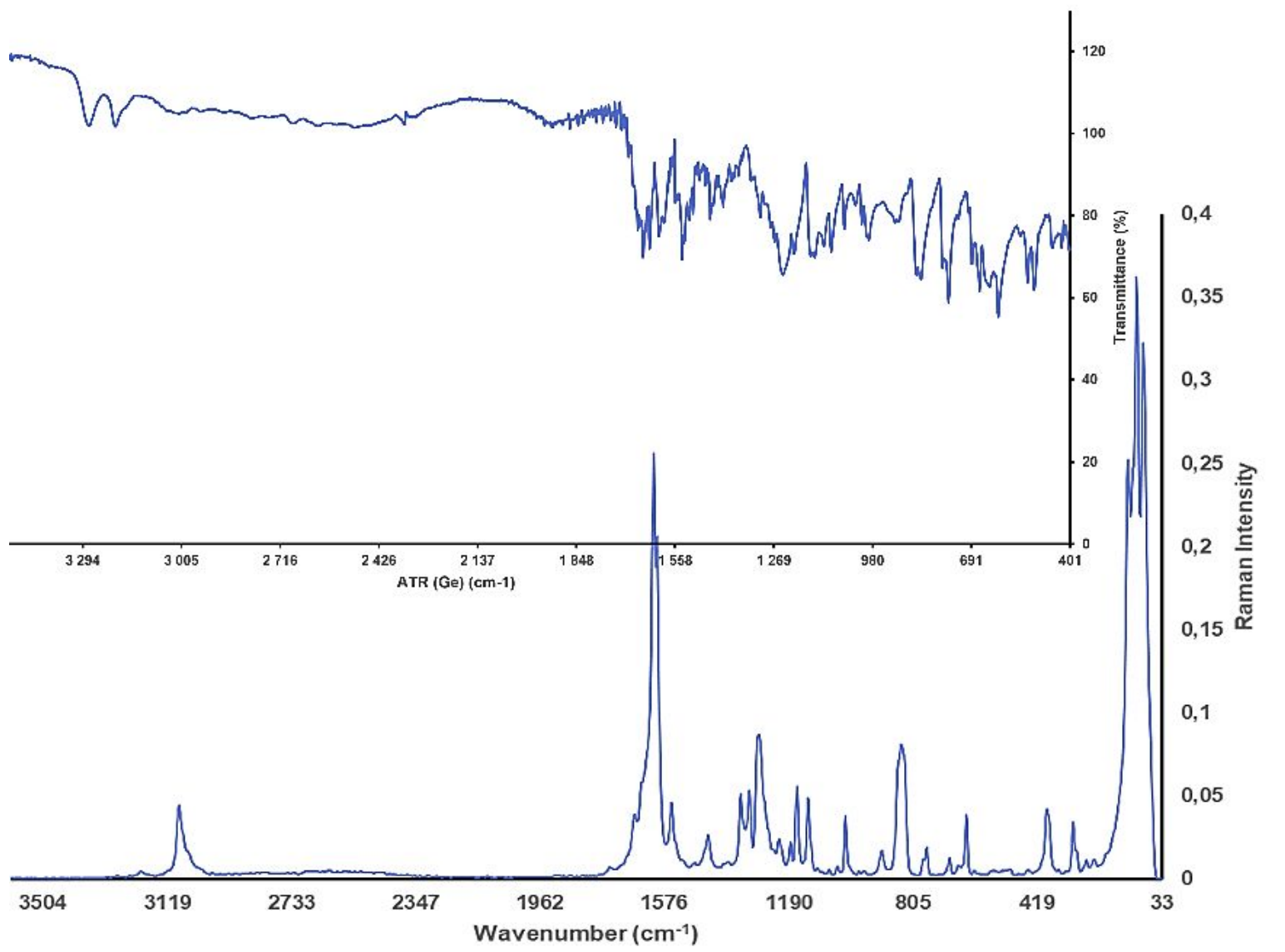

Figure S1: FTIR and Raman spectra for compound I (LabSolutions IR Version 2.26).

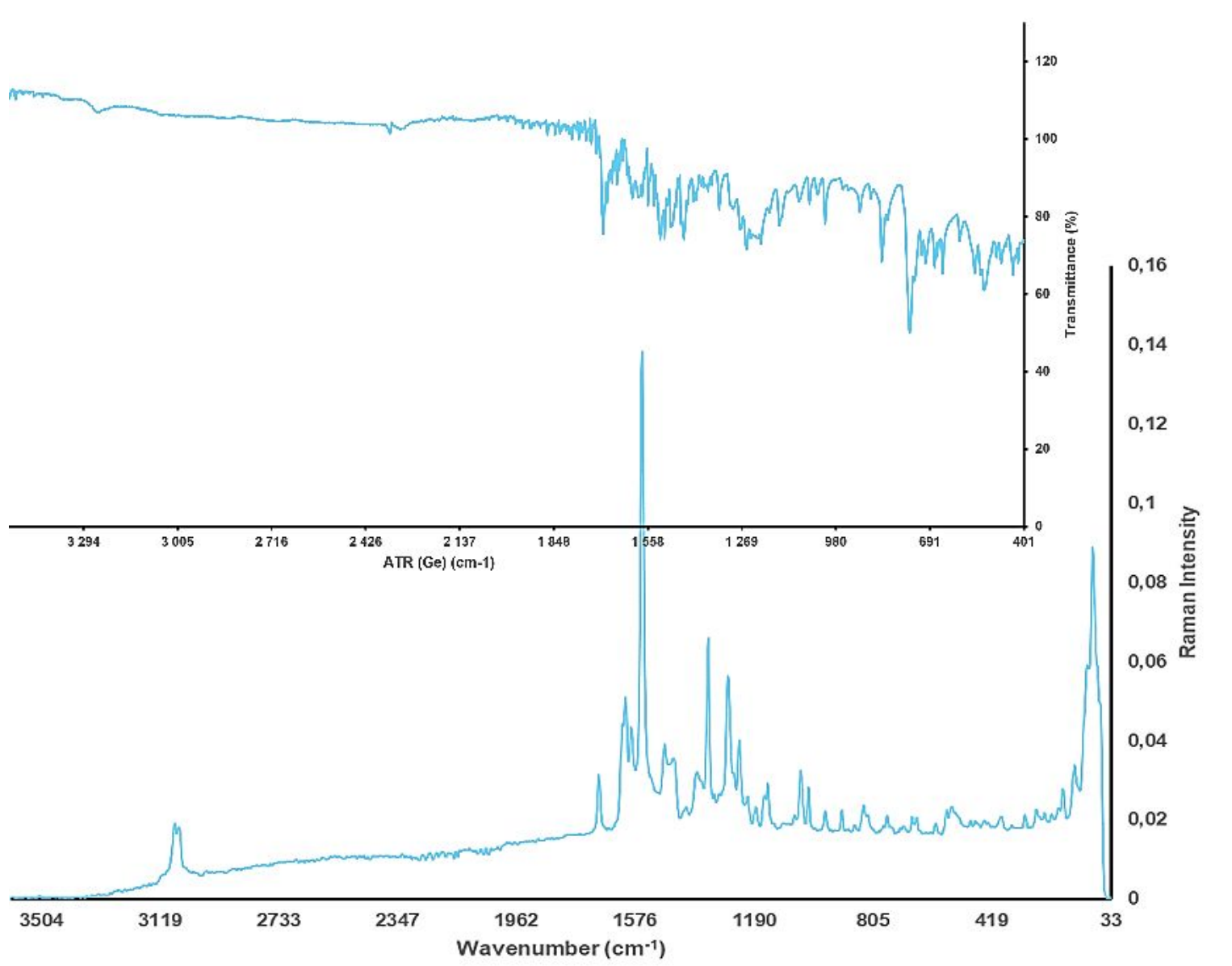

Figure S2: FTIR and Raman spectra for compound II (LabSolutions IR Version 2.26)

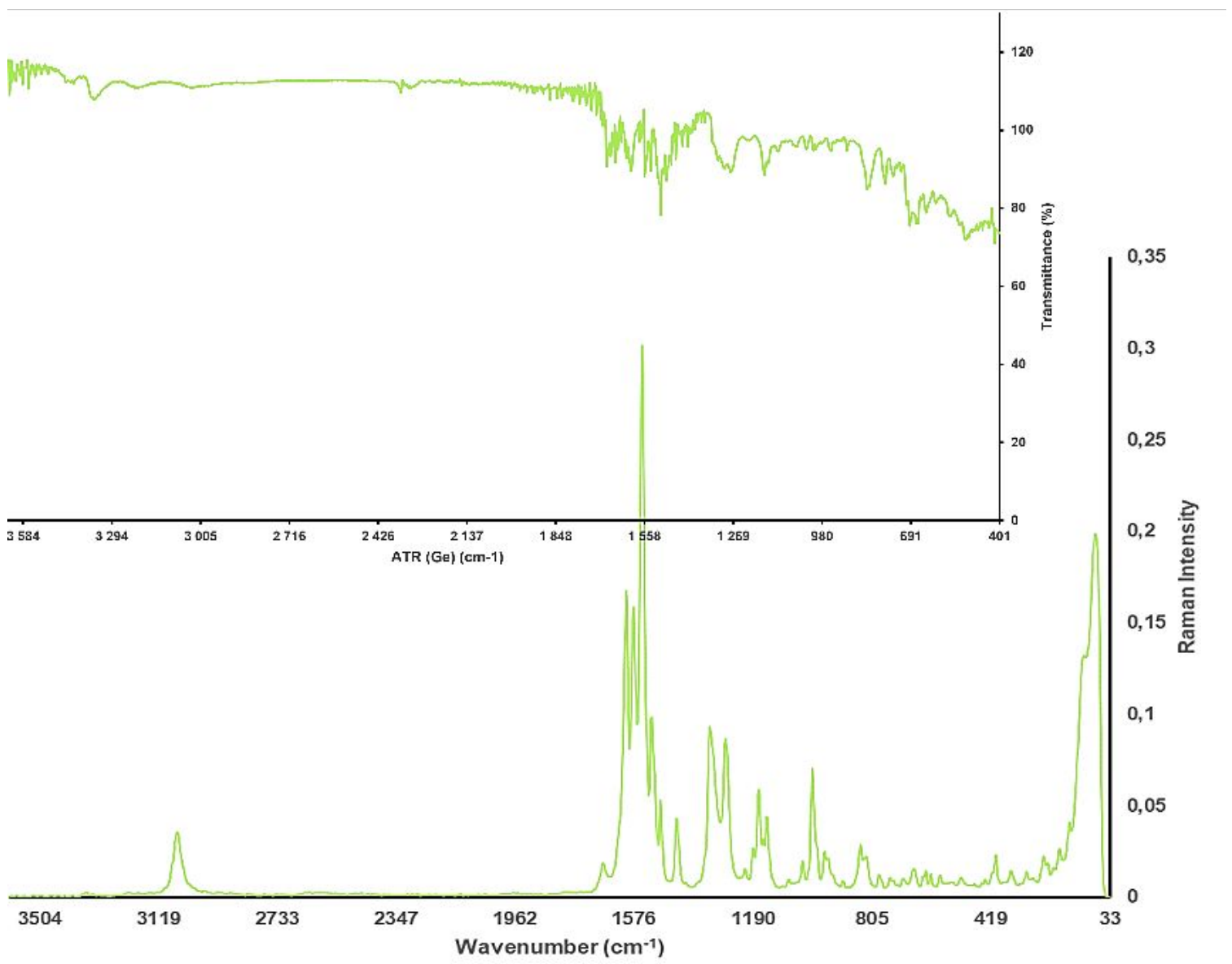

Figure S3: FTIR and Raman spectra for compound III

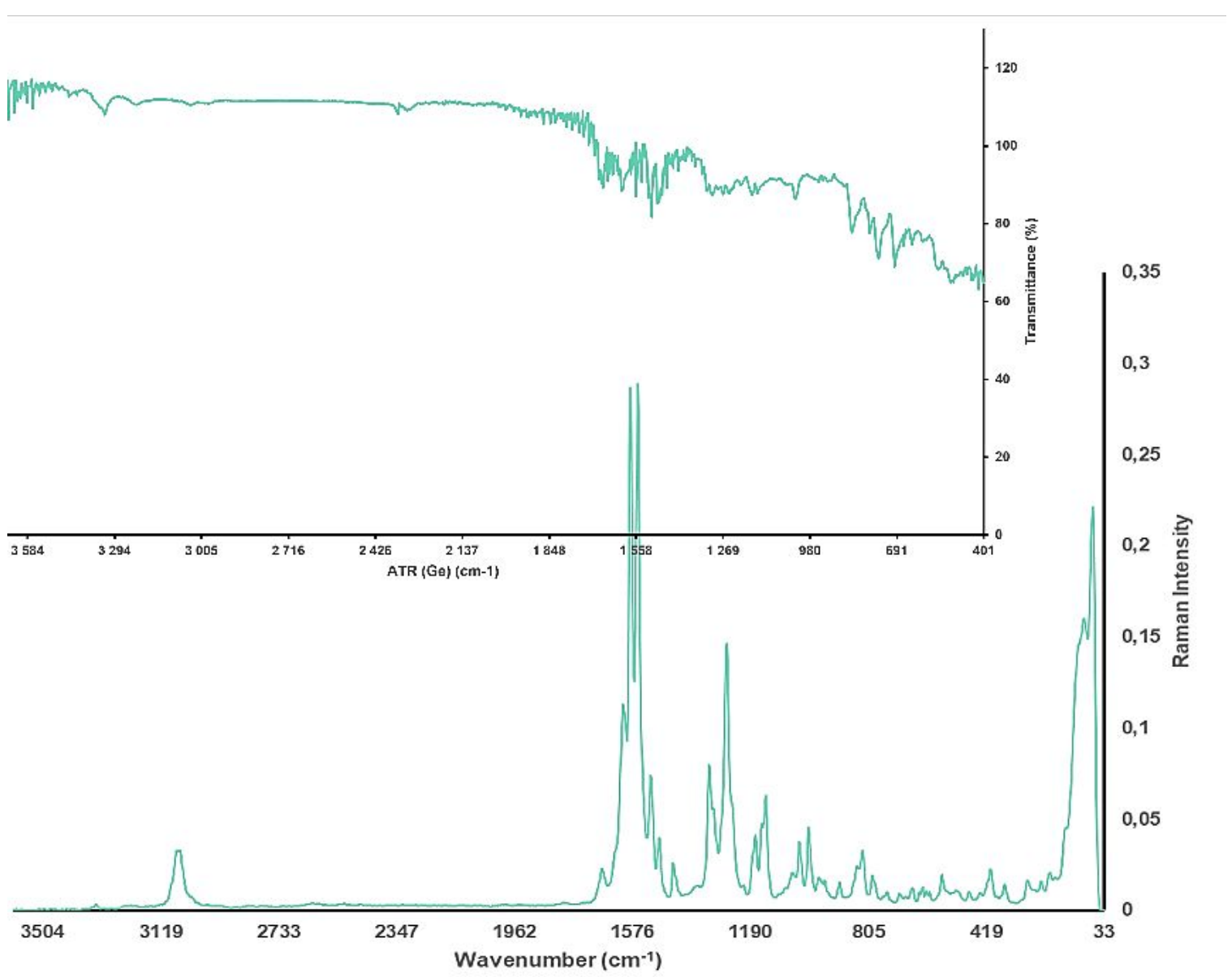

Figure S4: FTIR and Raman spectra for compound IV (LabSolutions IR Version 2.26)

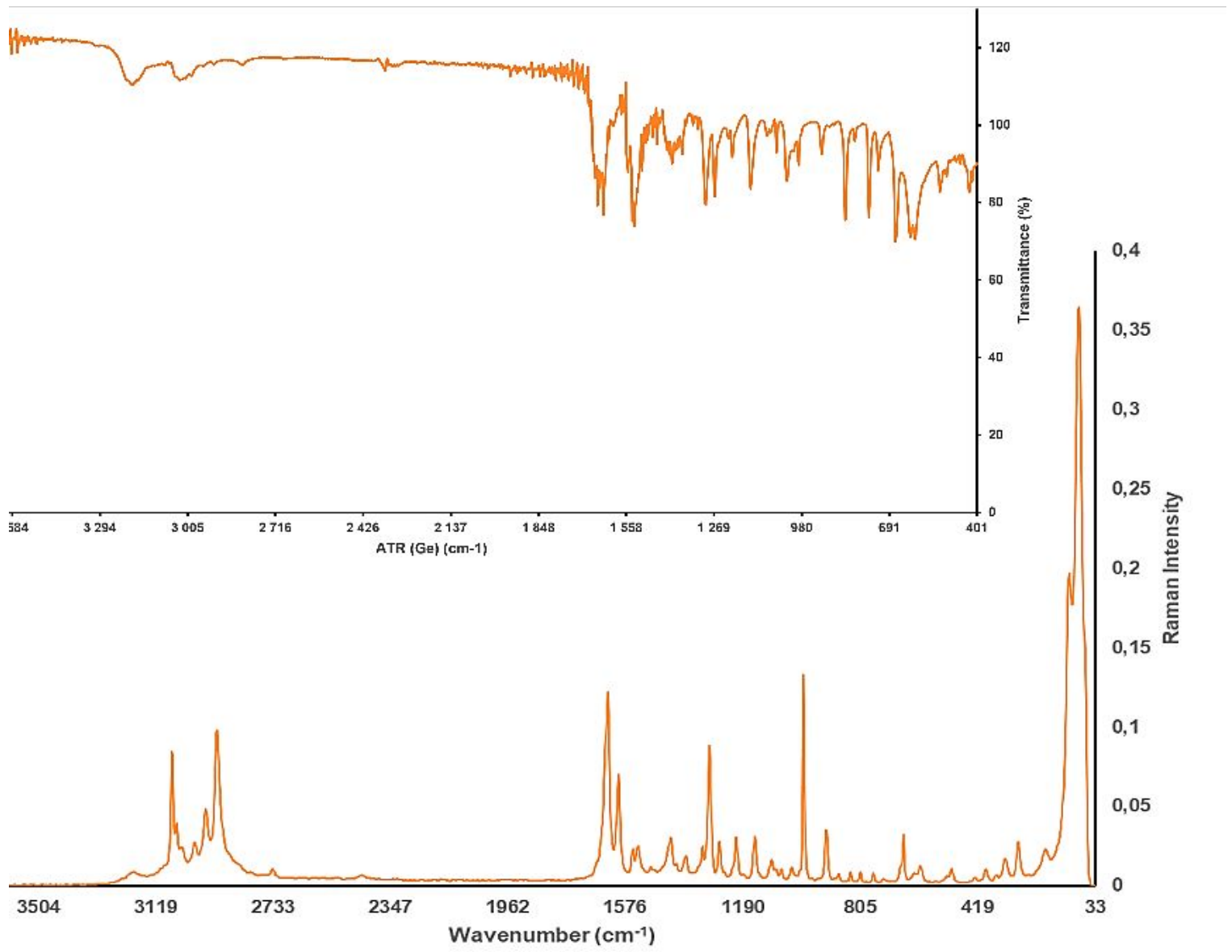

Figure S5: FTIR and Raman spectra for compound V

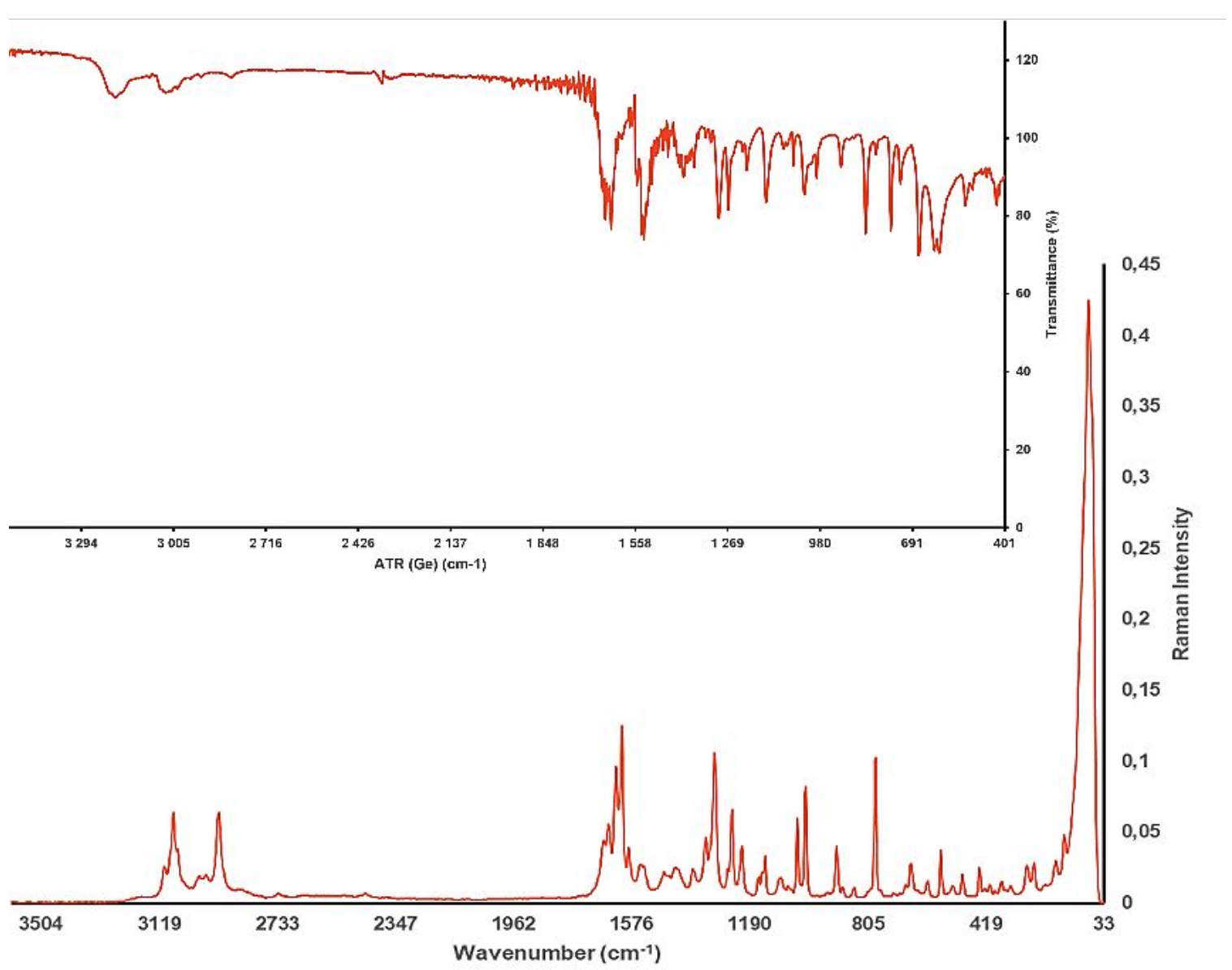

Figure S6: FTIR and Raman spectra for compound VI (LabSolutions IR Version 2.26)

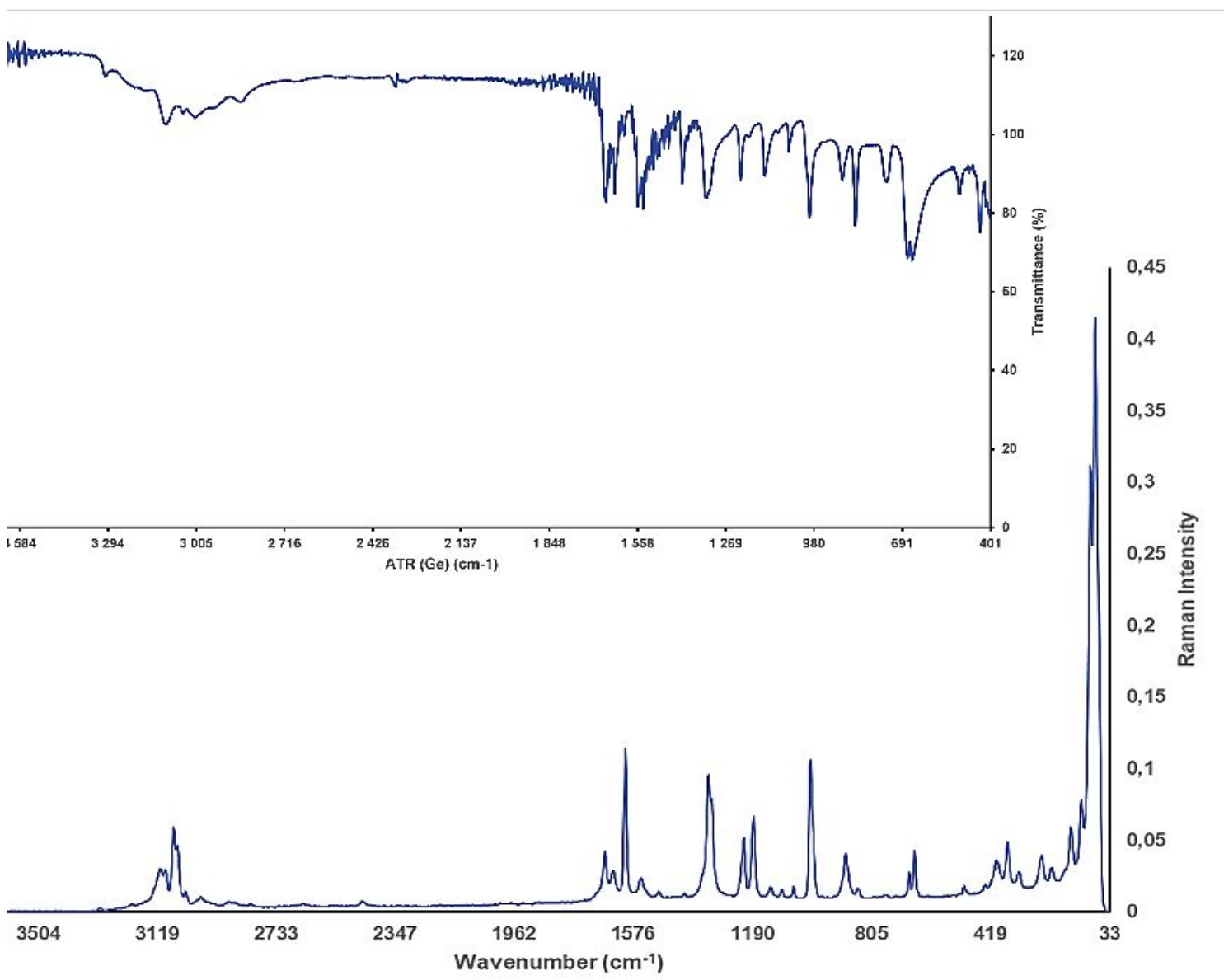

Figure S7: FTIR and Raman spectra for compound VII (LabSolutions IR Version 2.26).

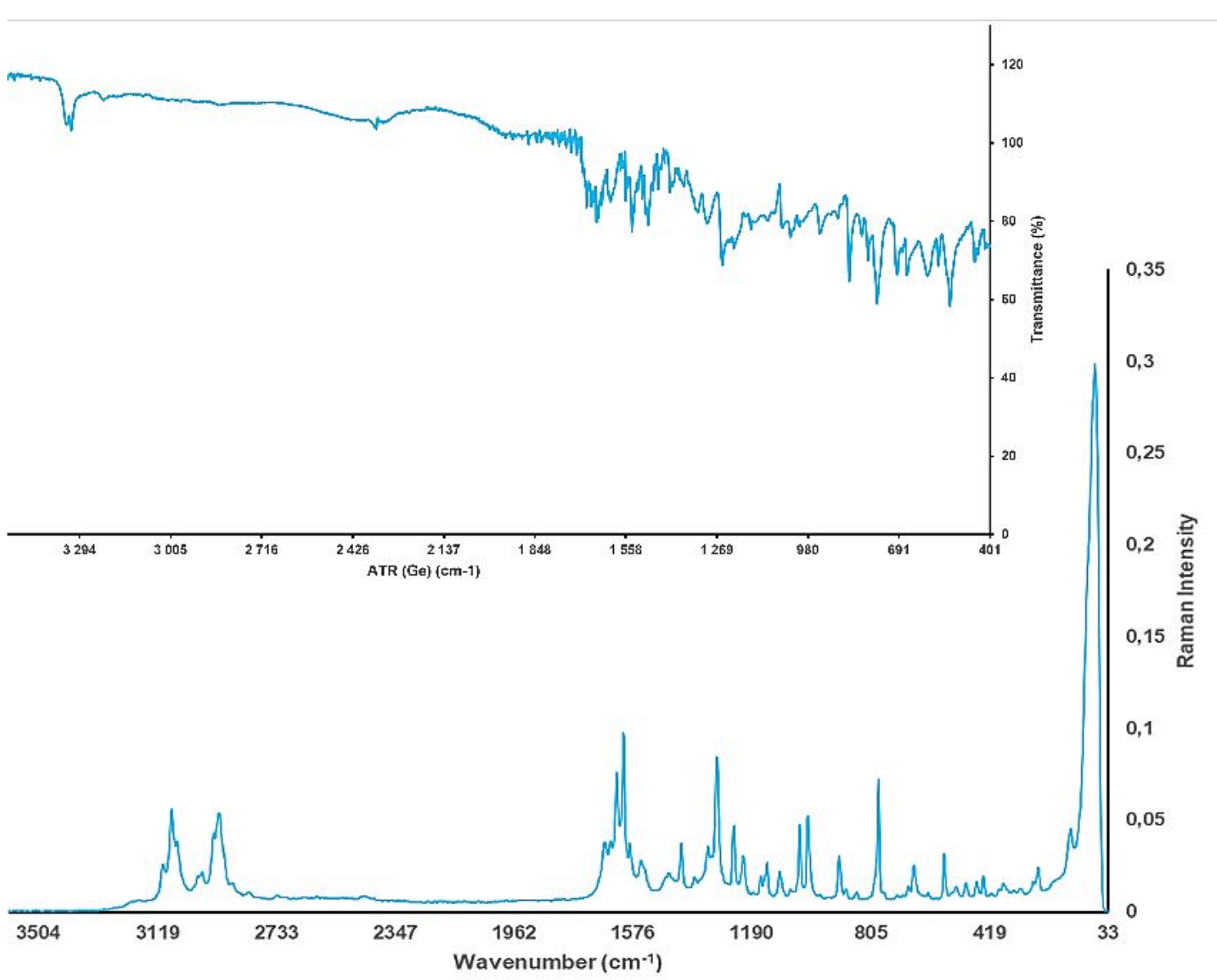

Figure S8: FTIR and Raman spectra for compound VIII (LabSolutions IR Version 2.26).

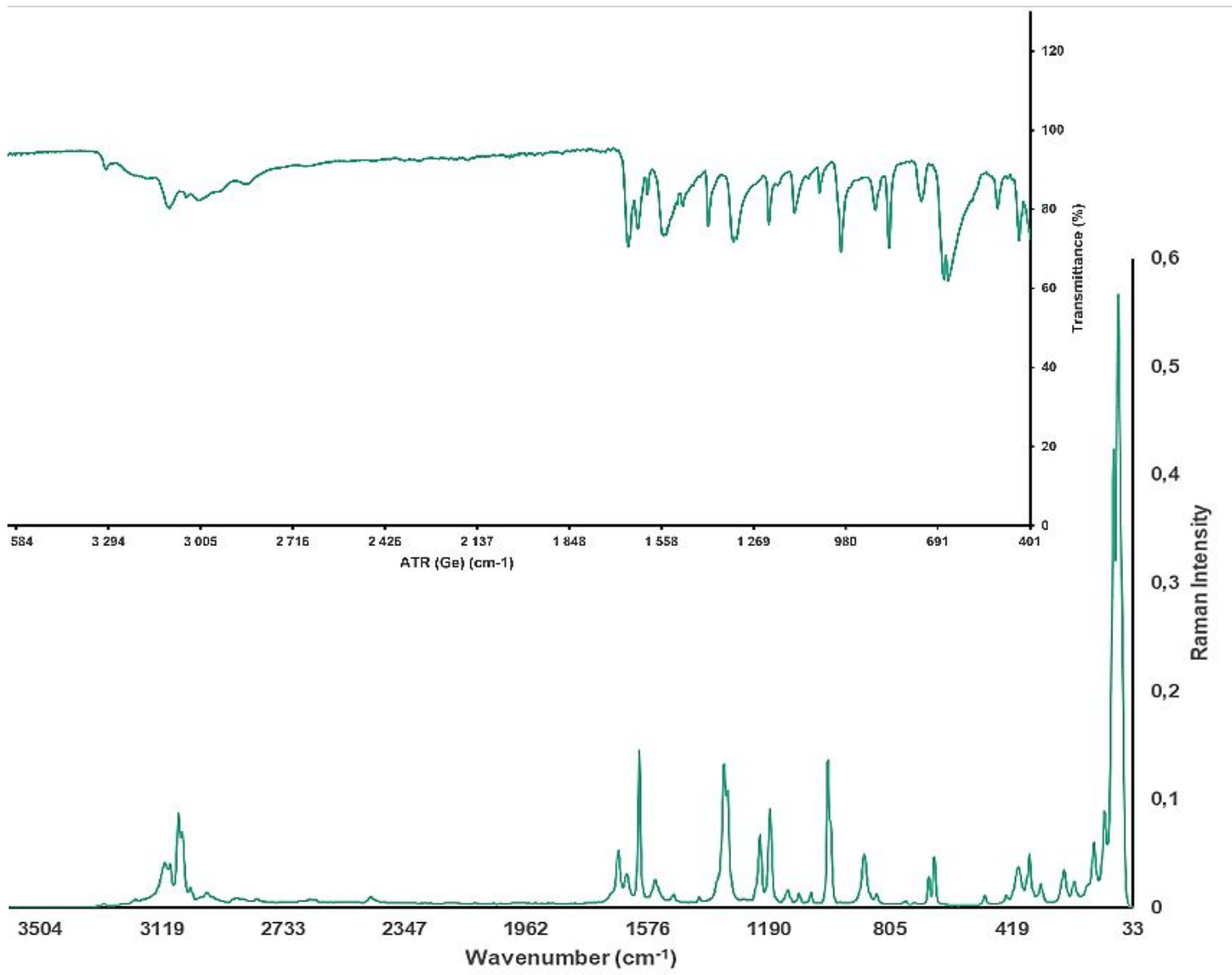

Figure S9: FTIR and Raman spectra for isoniazid (LabSolutions IR Version 2.26).

Table S2.1: ^{1}H NMR peak summary and compound positions for isoniazid

δH / ppm	Multiplicity/integration	Peak assignment	Position on compound
8.67-8.61	m, 2H	Pyridine aromatic protons	Py-H2/H6, nearest ring N
7.69-7.63	m, 2H	Pyridine aromatic protons	Py-H3/H5
4.7-4.9	br/s	HOD/water	Solvent, not compound
NH / NH_2	not clearly observed	Exchangeable protons	Hydrazide NH and NH2 exchange in D2O

Table S2.2: ^{1}H NMR peak summary and compound positions for compound I

δH / ppm	Multiplicity/integration	Peak assignment	Position on compound

~8.50-8.69	m/d	Pyridine protons	Py-H2/H6
~7.7-7.9	m	Pyridine/aryl protons	Py-H3/H5 and p-hydroxyphenyl H2/H6
~6.9-7.0	m	Phenolic aryl protons	p-Hydroxyphenyl H3/H5
OH / NH	mostly absent	Exchangeable protons	Phenolic OH and hydrazide NH in D2O
~4.8	s/br	HOD/water	Solvent

Table S2.3: ^{1}H NMR peak summary and compound positions for compound II

δH / ppm	Multiplicity/integration	Peak assignment	Position on compound
10.16	s/br	Possible NH/acidic proton or impurity	Exchangeable region; uncertain in D2O
8.60	d	Pyridine proton	Py-H2/H6
7.79	dd/overlap	Pyridine + salicylate overlap	Py-H3/H5 and/or Sal-H6
7.55-7.25	m	Salicylate aromatic protons	Mainly Sal-H4/H6
7.26-7.08	m	Hydrazone aryl protons	p-Hydroxyphenyl aromatic H
7.03-6.82	m	Aryl + salicylate overlap	p-Hydroxyphenyl H and Sal-H3/H5
6.79-6.64	m	Salicylate/phenolic aryl protons	Sal-H3/H5
~4.8	s/br	HOD/water	Solvent
3.36, 2.23, 2.19, 1.92	small	Minor impurities/residual solvent	Not assigned to product

Table S2.4: ^{1}H NMR peak summary and compound positions for compound III

δH / ppm	Multiplicity/integration	Peak assignment	Position on compound
~8.6	m/d	Pyridine protons	Isoniazid Py-H2/H6
~7.8-7.5	m	Pyridine + p-hydroxybenzoate protons	Py-H3/H5 and p-HBA H2/H6
~6.9-6.7	m	p-Hydroxybenzoate protons	p-HBA H3/H5
~4.8	s/br	HOD/water	Solvent

Table S2.5: ^{1}H NMR peak summary and compound positions for compound IV

δH / ppm	Multiplicity/integration	Peak assignment	Position on compound
8.69-8.59	m, ~2H	Pyridine protons	Py-H2/H6
7.83	dd/overlap	Pyridine/aryl overlap	Py-H3/H5, phenyl H, salicylate H
7.72	dd/overlap	Salicylate/aryl overlap	Sal-H6 and hydrazone aryl H
7.51-7.42	m	Phenyl + salicylate protons	Hydrazone phenyl H and Sal-H4
7.02-6.92	m	Aminophenyl + salicylate protons	4-Aminophenyl H and Sal-H3/H5
3.36	s	Residual solvent/impurity	Not product
~4.8	s/br	HOD/water	Solvent
NH2 / NH / OH / COOH	mostly absent	Exchangeable protons	Exchangeable in D2O

Table S2.6: ^{1}H NMR peak summary and compound positions for compound V

δH / ppm	Multiplicity/integration	Peak assignment	Position on compound
8.58-8.52	m, 2H	Pyridine protons	Py-H2/H6
7.77-7.62	m, 2H	Pyridine protons	Py-H3/H5
2.05	s, 3H	Hydrazone methyl	C=N-C(CH3)2, Me-a
1.88	s, 3H	Hydrazone methyl	C=N-C(CH3)2, Me-b
2.23	s	Residual acetone/free acetone	Impurity
4.81	s	HOD/water	Solvent
0.00	s	Reference	TMS/reference

Table S2.7: ^{1}H NMR peak summary and compound positions for compound VI

δH / ppm	Multiplicity/integration	Peak assignment	Position on compound
8.80-8.72	m	Pyridine protons	Hydrazone Py-H2/H6
7.98-7.90	m	Pyridine + salicylate overlap	Py-H3/H5 and salicylate H
7.92-7.86	m	Salicylate aromatic proton	Sal-H6, near CO2H/CO2-
7.75	dd, J = 7.9, 1.8 Hz	Salicylate aromatic proton	Sal-H3/H5/H6, ortho-coupled
7.41	ddd, J = 8.2, 7.3, 1.8 Hz	Salicylate aromatic proton	Sal-H4
6.96-6.84	m	Salicylate aromatic protons	Sal-H3/H5
2.22-2.06	m/s region	Hydrazone methyls	C=N-C(CH3)2 methyl groups
~4.90	s/br	HOD/water	Solvent
0.00	s	Reference	TMS/reference

Table S2.8: ^{1}H NMR peak summary and compound positions for compound VII

δH / ppm	Multiplicity/integration	Peak assignment	Position on compound
8.67-8.61	m	Pyridine protons	Py-H2/H6
7.69-7.63	m	Pyridine protons	Py-H3/H5
4.68	s/br	HOD/water	Hydrate/solvent water
2.01, 1.18, 0.96	trace	Trace impurities	Too small for hydrazone alkyl groups

Table S2.9: ^{1}H NMR peak summary and compound positions for compound VIII

δH / ppm	Multiplicity/integration	Peak assignment	Position on compound
8.84-8.71	m	Pyridine protons	Py-H2/H6
7.90-7.84	m	Pyridine protons	Py-H3/H5
7.78	dd, J = 7.8, 1.8 Hz	Salicylate aromatic proton	Sal-H6 or H3/H5
7.44	ddd, J = 8.5, 7.3, 1.8 Hz	Salicylate aromatic proton	Sal-H4
6.99-6.87	m	Salicylate aromatic protons	Sal-H3/H5
2.58, 2.20-2.09, 1.17-0.98	small/weak	Weak aliphatic signals	Not reliable as product butanone hydrazone signals
4.83	s/br	HOD/water	Solvent

Table S3.1: ^{13}C NMR peak summary and compound positions for isoniazid

δC / ppm	Peak assignment	Position on compound
166.62	Hydrazide carbonyl	CONH-NH2 C=O
149.37	Pyridine carbons near N	Py-C2/C6
141.07	Pyridine ipso carbon	Py-C4-C(O)
121.47	Pyridine CH carbons	Py-C3/C5

Table S3.2: ^{13}C NMR peak summary and compound positions for compound I

δC / ppm	Peak assignment	Position on compound
159.63	Phenolic C-O; possible overlap with C=N/C=O	p-Hydroxyphenyl C-OH
148.66	Pyridine carbons near N	Py-C2/C6
131.73	Aromatic CH	p-Hydroxyphenyl C2/C6
121.96	Pyridine CH carbons	Py-C3/C5
115.06	Phenolic aromatic CH	p-Hydroxyphenyl C3/C5

Table S3.3: ^{13}C NMR peak summary and compound positions for compound III

δC / ppm	Peak assignment	Position on compound
149.19	Pyridine carbons near N	Isoniazid Py-C2/C6
129.41	Aromatic CH	p-Hydroxybenzoate aryl carbon
121.74	Pyridine/aryl CH overlap	Py-C3/C5 and/or benzoate CH

Table S3.4: ^{13}C NMR peak summary and compound positions for compound IV

δC / ppm	Peak assignment	Position on compound
133.94	Aromatic carbon	Phenyl/salicylate aryl carbon
130.45	Aromatic CH	Phenyl/salicylate CH
119.33	Aromatic CH	Aminophenyl/salicylate CH
116.25	Aromatic CH	Aminophenyl/salicylate CH

Table S3.5: ^{13}C NMR peak summary and compound positions for compound V

δC / ppm	Peak assignment	Position on compound
163.57	Hydrazide carbonyl	CONH-N=C C=O
162.87	Hydrazone imine carbon	C=N
148.83, 148.70	Pyridine carbons near N	Py-C2/C6
122.11, 121.33	Pyridine CH carbons	Py-C3/C5
23.85	Hydrazone methyl carbon	Me-a of C=N-C(CH3)2
17.15	Hydrazone methyl carbon	Me-b of C=N-C(CH3)2

Table S3.6: ^{13}C NMR peak summary and compound positions for compound VI

δC / ppm	Peak assignment	Position on compound
174.75	Salicylate carbonyl	Sal-CO2H/CO2-

169.29	Hydrazide carbonyl	CONH-N=C C=O
165.62	Hydrazone imine carbon	C=N
159.51	Phenolic carbon	Sal-C2-OH
146.64, 146.57	Pyridine carbons near N	Py-C2/C6
144.02, 143.52	Pyridine/quaternary aromatic carbons	Py-C4 and/or salicylate ipso
134.14, 130.38	Salicylate aromatic CH	Salicylate H-bearing carbons
123.21, 122.89, 122.21	Pyridine/aromatic CH	Py-C3/C5 and overlapping aryl carbons
119.30, 117.03, 116.24	Salicylate aromatic CH	Sal-C3/C5-type carbons
24.07	Hydrazone methyl carbon	Me-a of C=N-C(CH3)2
17.94	Hydrazone methyl carbon	Me-b of C=N-C(CH3)2
215.24, 30.15	Residual acetone	Free acetone C=O and CH3; not product

Table S3.7: ^{13}C NMR peak summary and compound positions for compound VII

δC / ppm	Peak assignment	Position on compound
166.62	Hydrazide carbonyl	Isoniazid CONHNH2 C=O
149.37	Pyridine carbons near N	Py-C2/C6
141.07	Pyridine ipso carbon	Py-C4-C(O)
121.47	Pyridine CH carbons	Py-C3/C5
Expected but absent	C=N-CH3, C=N-CH2CH3, terminal CH3	Required hydrazone alkyl carbons below 50 ppm are not observed

Table S3.8: ^{13}C NMR peak summary and compound positions for compound VIII

δC / ppm	Peak assignment	Position on compound
175.05	Salicylate carbonyl	Sal-CO2H/CO2-
166.10	Hydrazide carbonyl, possible C=N overlap	CONH / C=N region
159.54	Phenolic carbon	Sal-C2-OH
147.09	Pyridine carbons near N	Py-C2/C6
143.22	Pyridine ipso carbon	Py-C4-C(O)
134.13, 130.43	Salicylate aromatic CH	Salicylate H-bearing carbons
122.74	Pyridine CH carbons	Py-C3/C5
119.35, 117.30, 116.27	Salicylate aromatic CH	Sal-C3/C5-type carbons
Expected but absent	C=N-CH3, C=N-CH2CH3, terminal CH3	Required butanone hydrazone alkyl carbons below 50 ppm are not clear

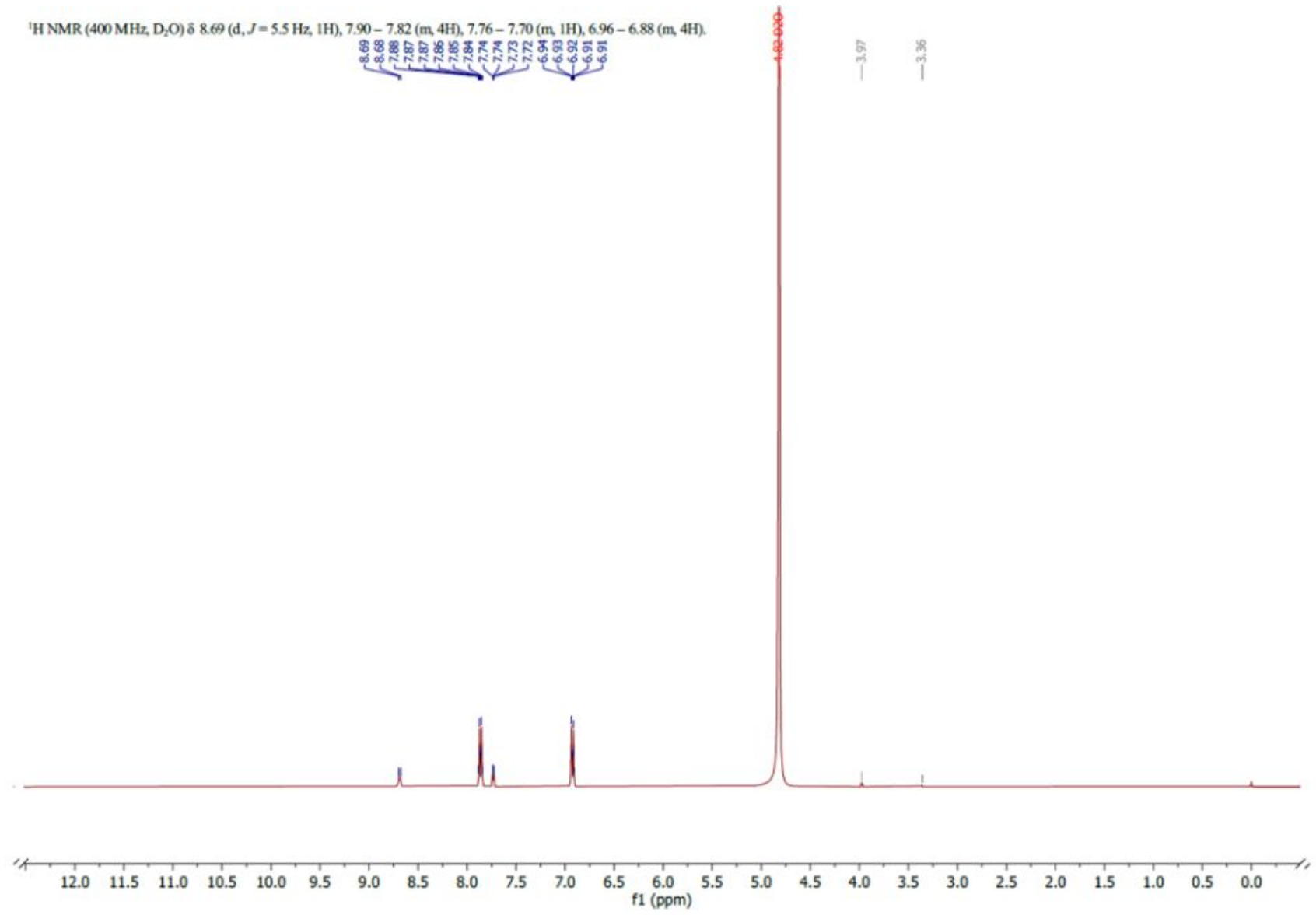

Figure S10: ^{1}H NMR spectra of compound I recorded in deuterated water at room temperature (MestReNova Version 15.0.1-35756).

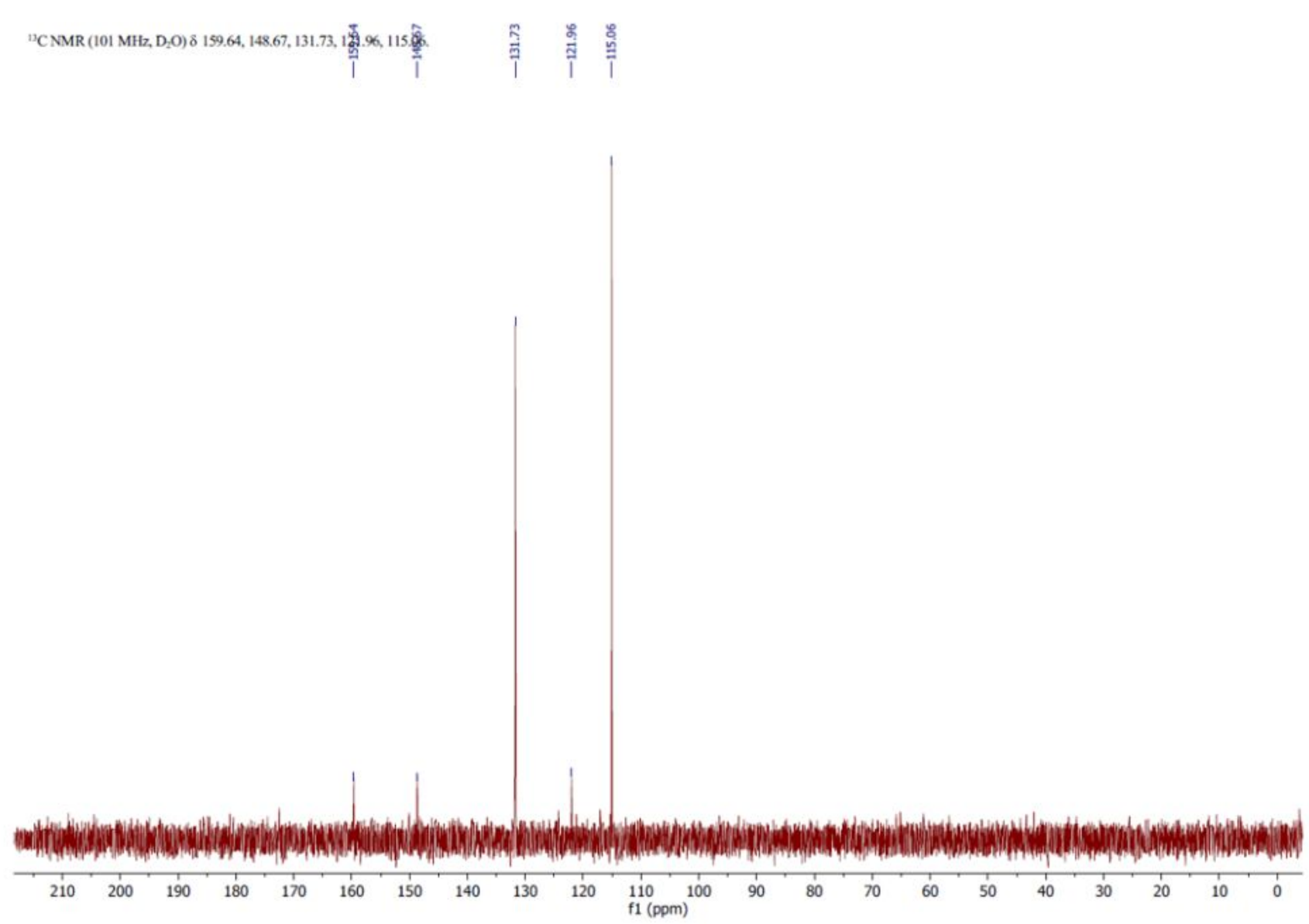

Figure S11: ^{13}C NMR spectra of compound I recorded in deuterated water at room temperature (MestReNova Version 15.0.1-35756).

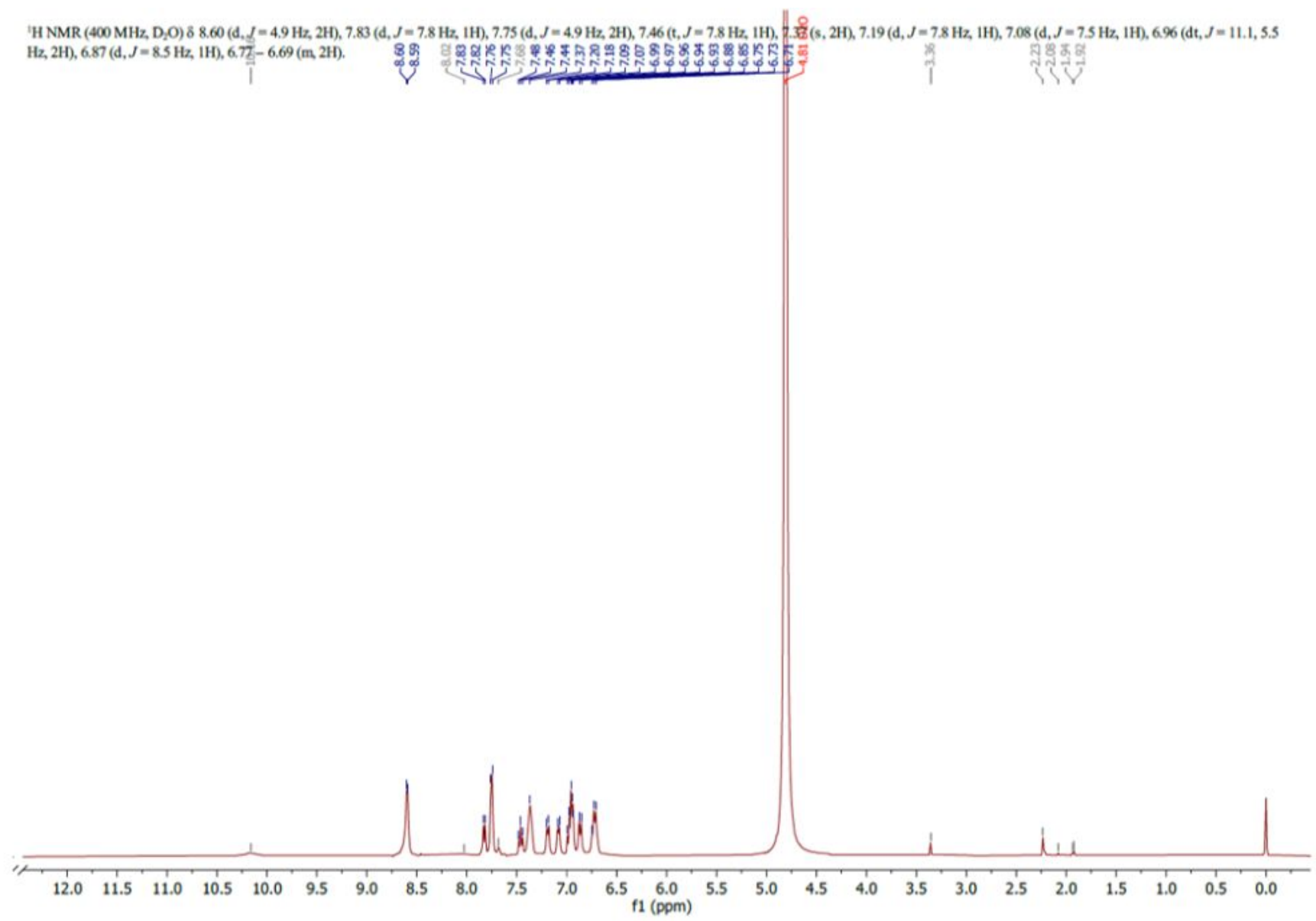

Figure S12: ^{1}H NMR spectra of compound II recorded in deuterated water at room temperature (MestReNova Version 15.0.1-35756).

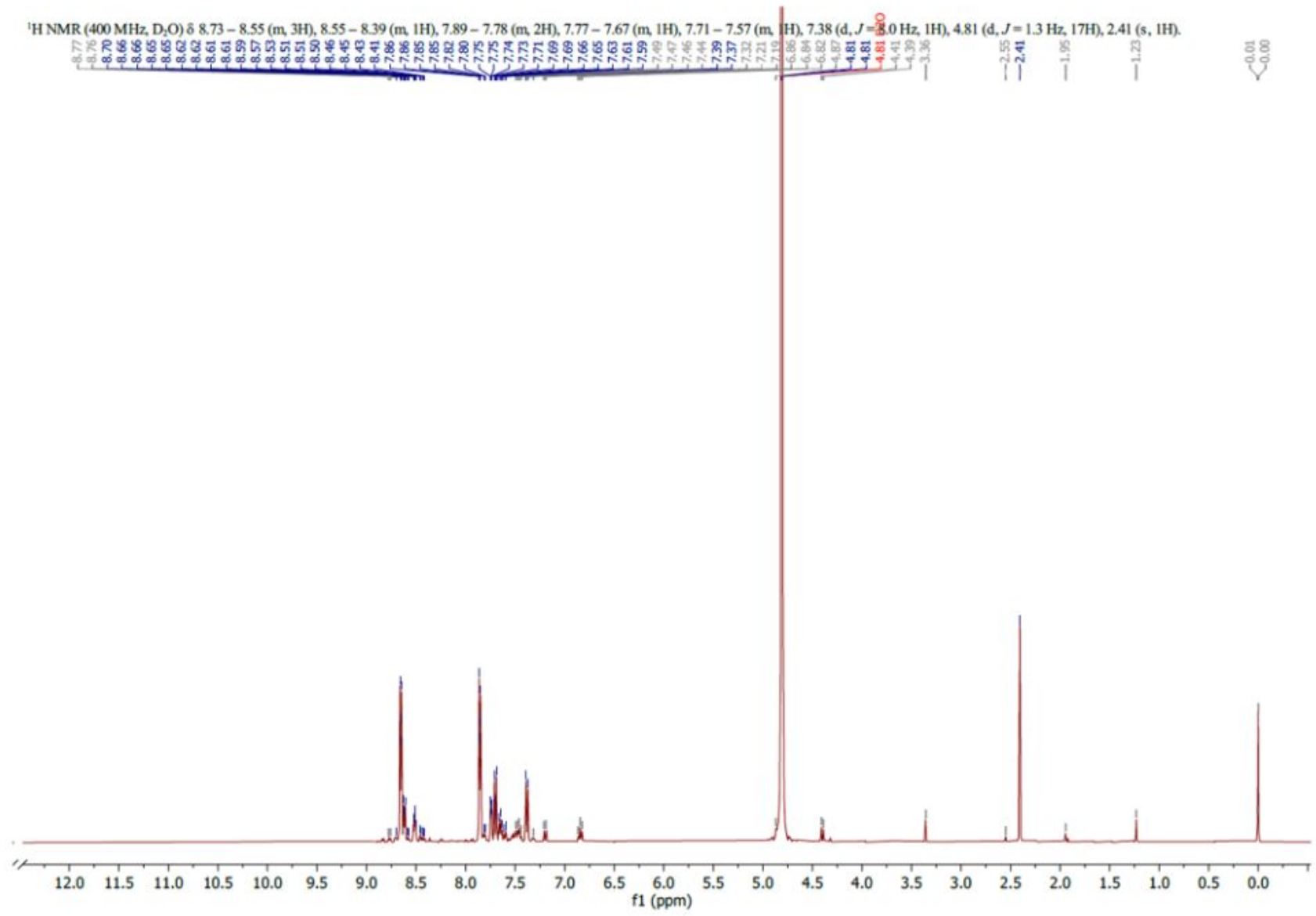

Figure S13: ^{1}H NMR spectra of compound III recorded in deuterated water at room temperature (MestReNova Version 15.0.1-35756).

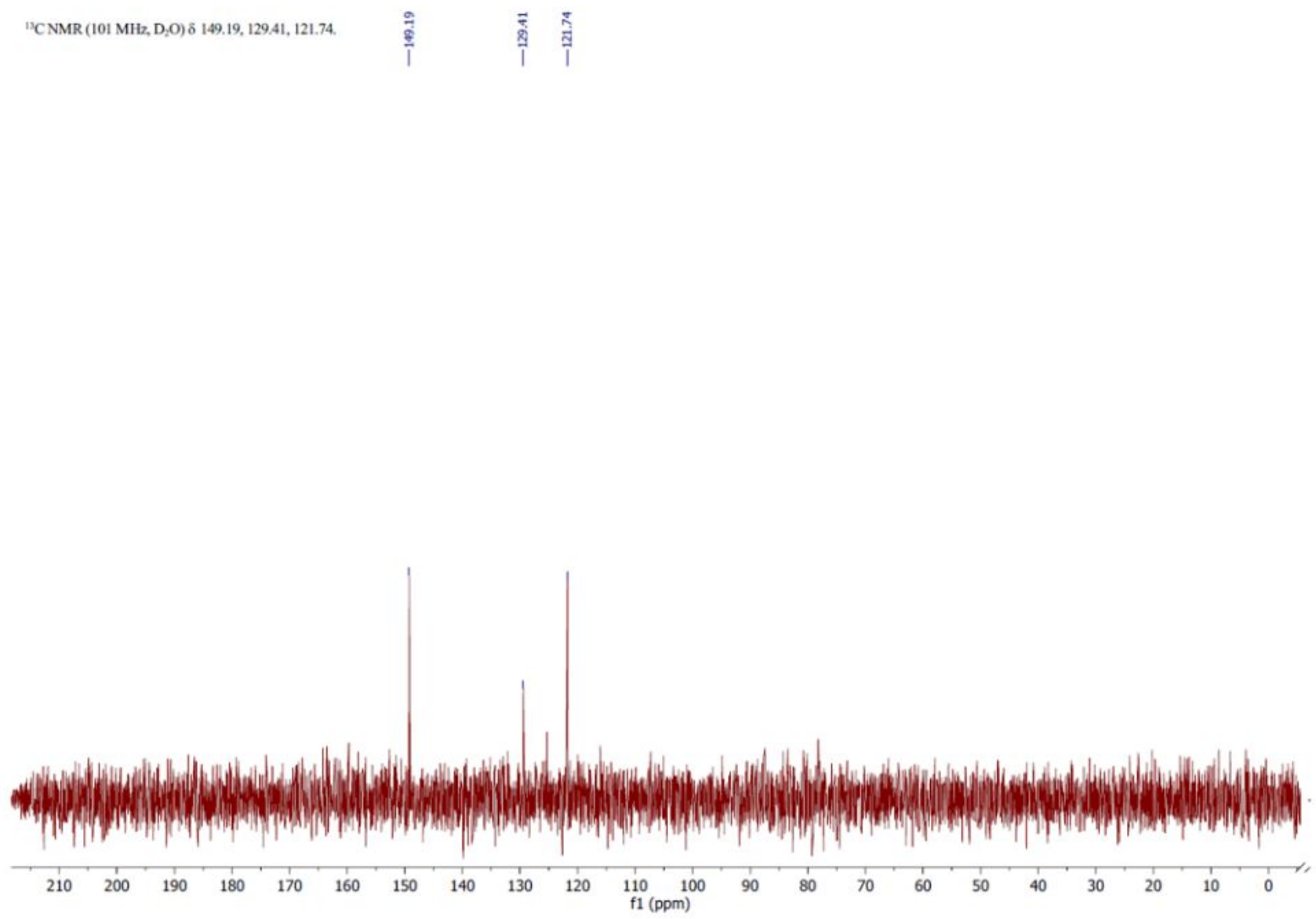

Figure S14: ^{13}C NMR spectra of compound III recorded in deuterated water at room temperature (MestReNova Version 15.0.1-35756).

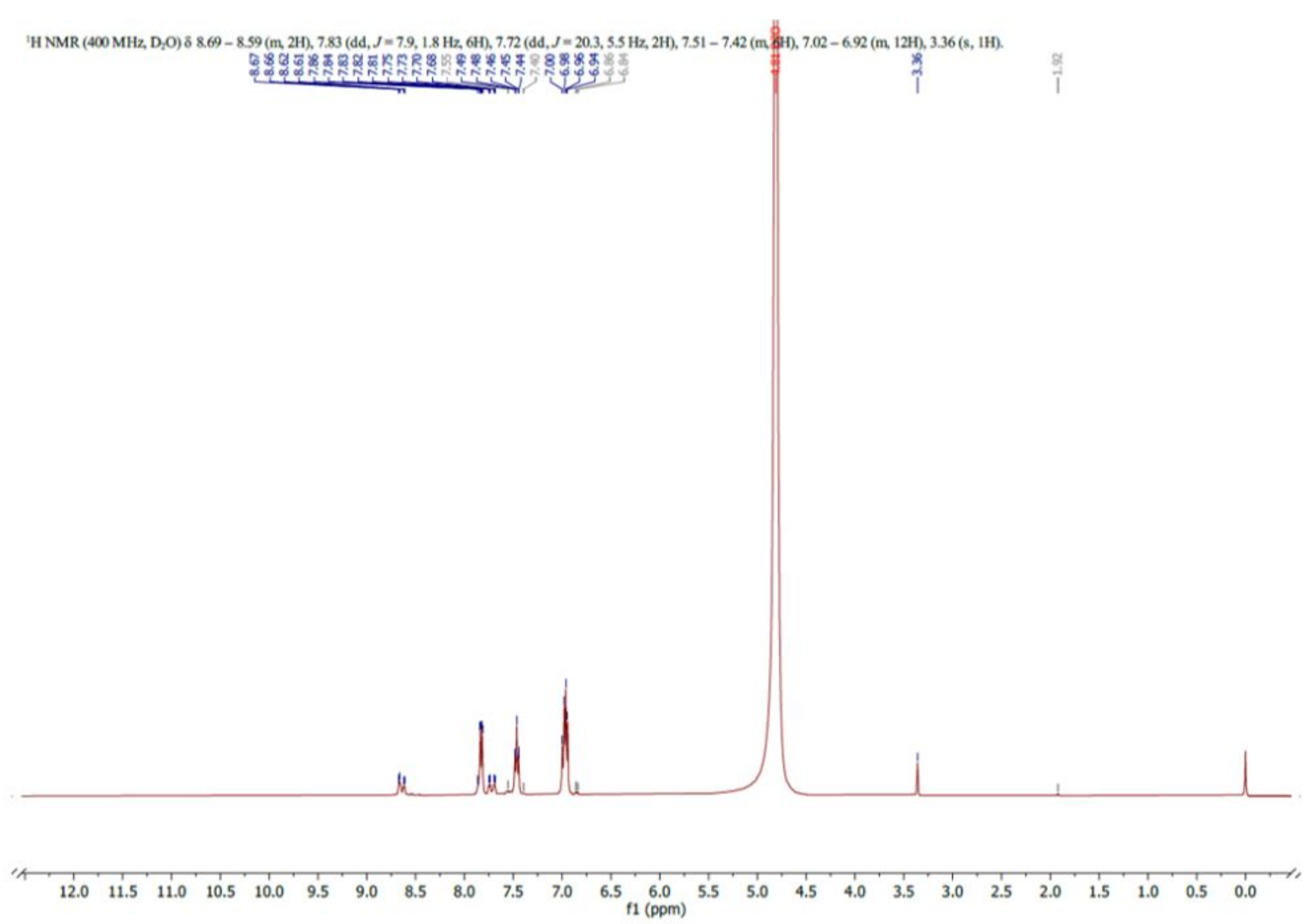

Figure S15: ^{1}H NMR spectra of compound IV recorded in deuterated water at room temperature (MestReNova Version 15.0.1-35756).

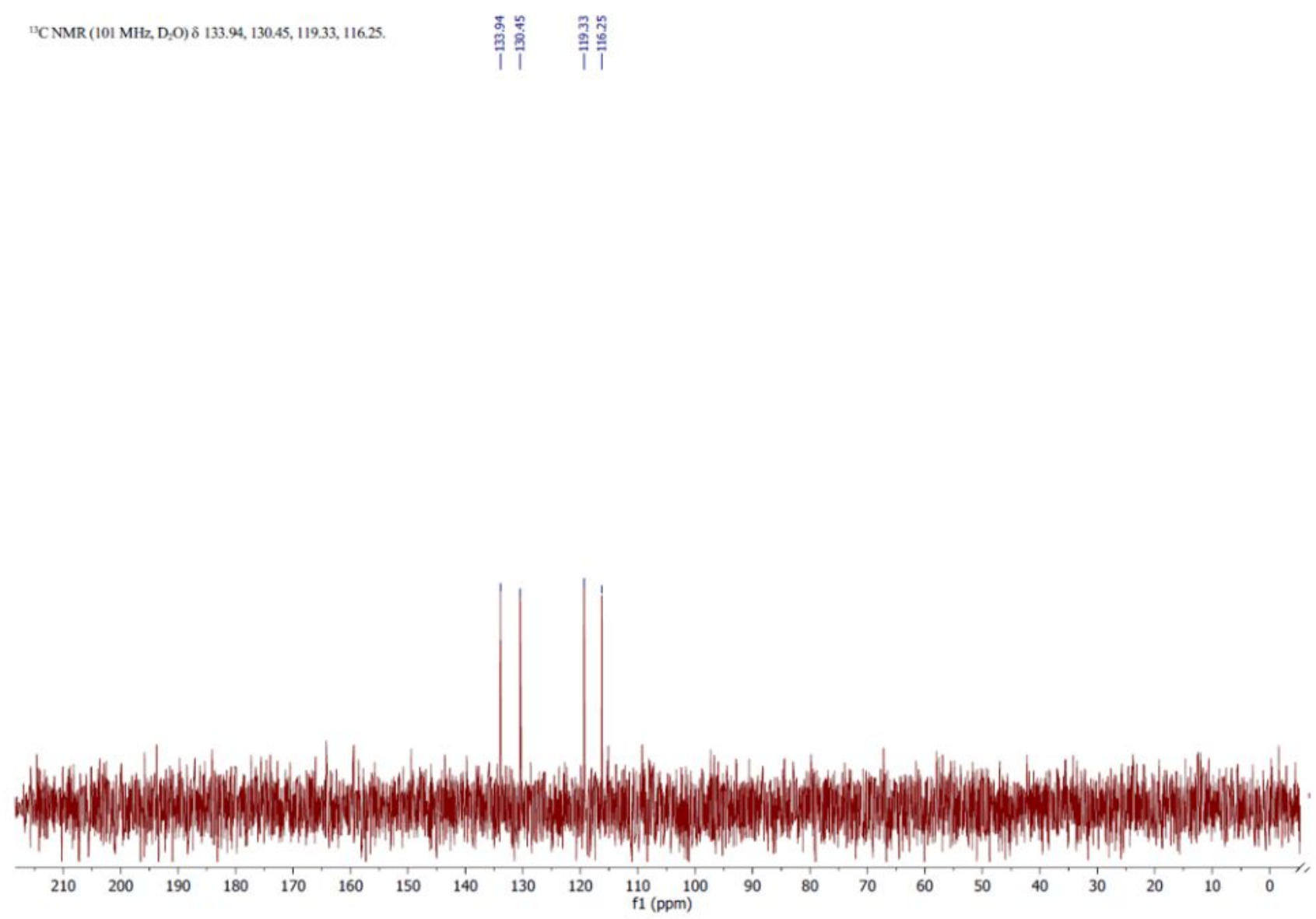

Figure S16: ^{13}C NMR spectra of compound IV recorded in deuterated water at room temperature (MestReNova Version 15.0.1-35756).

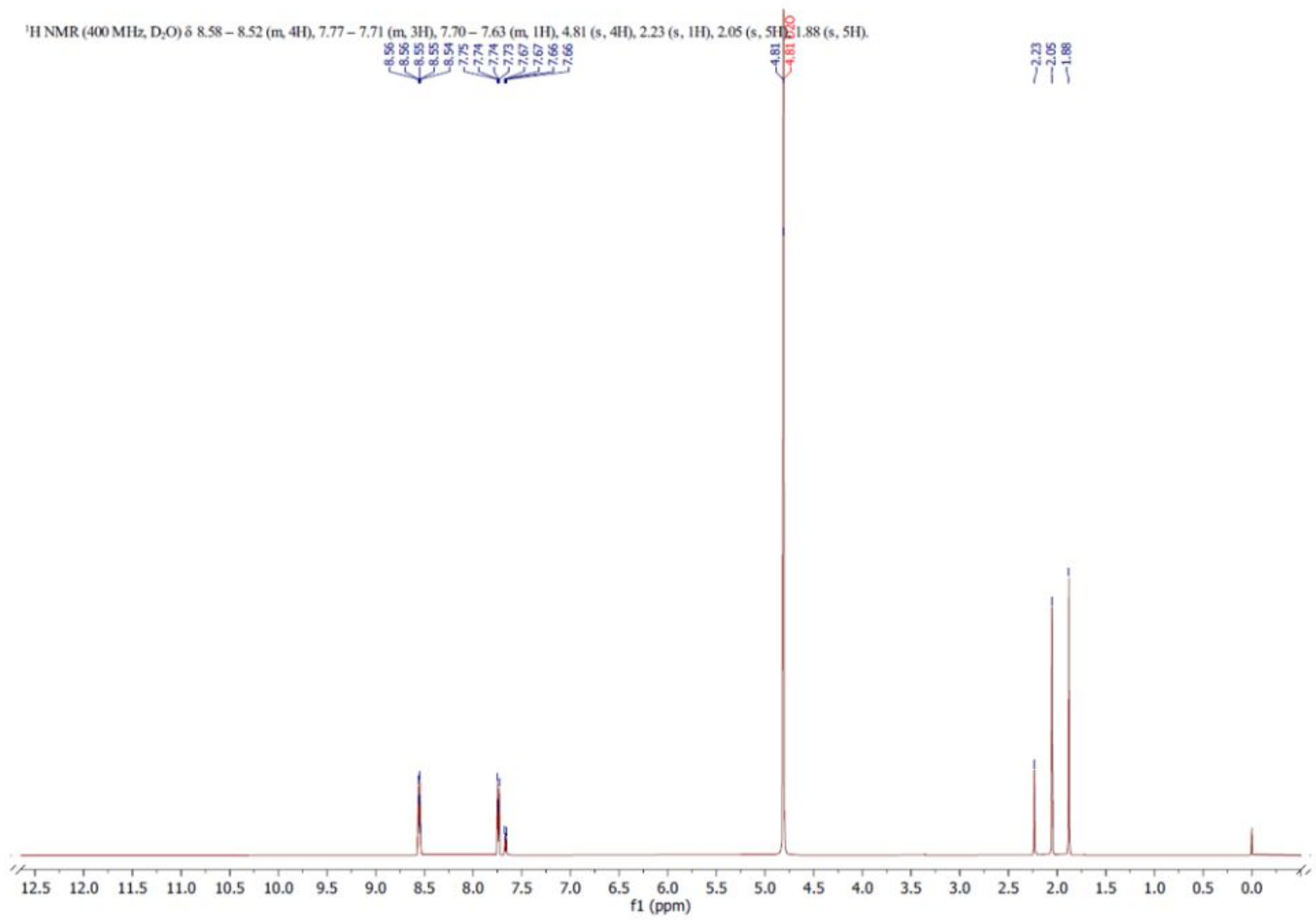

Figure S17: ^{1}H NMR spectra of compound V recorded in deuterated water at room temperature (MestReNova Version 15.0.1-35756).

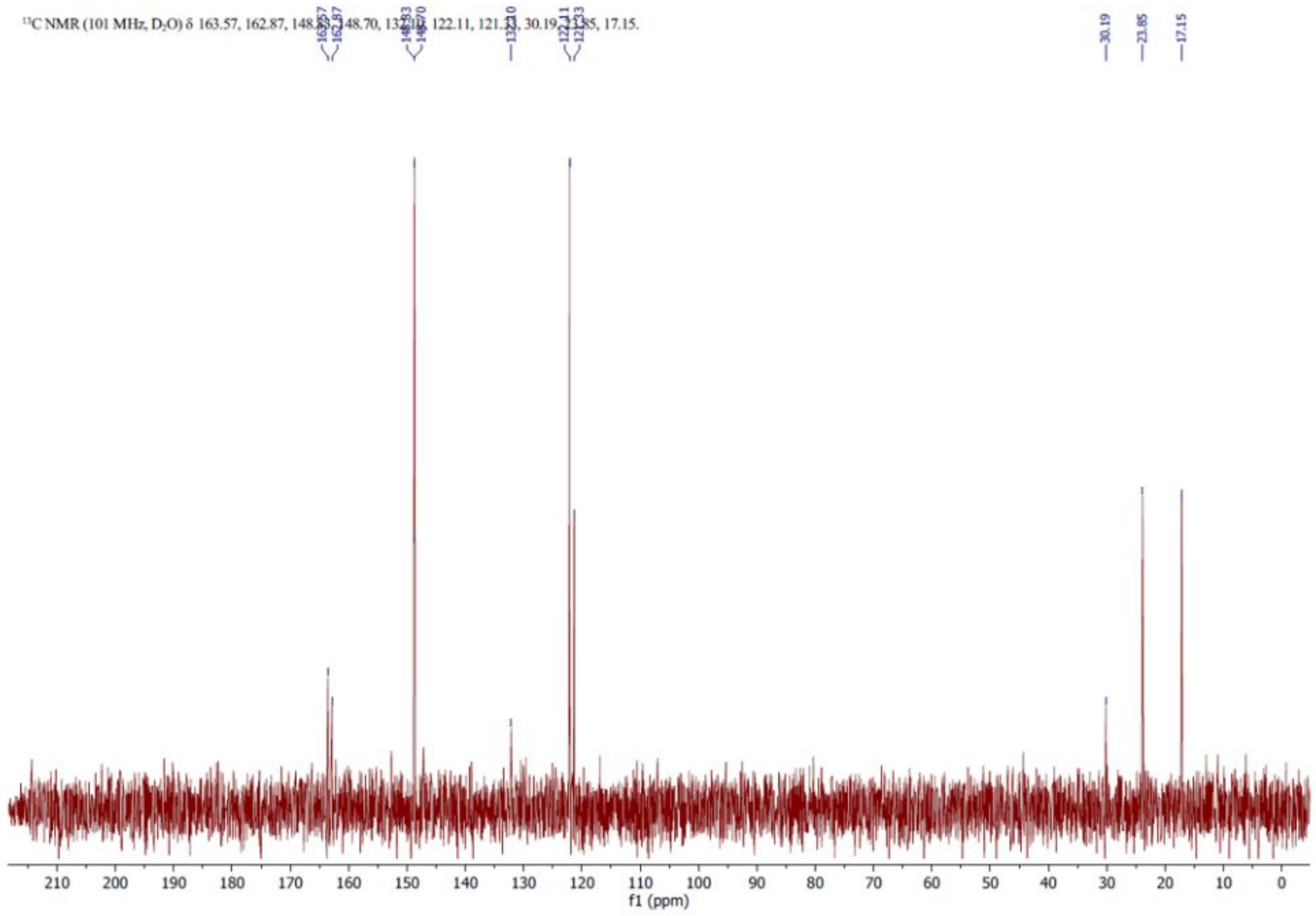

Figure S18: ^{13}C NMR spectra of compound V recorded in deuterated water at room temperature (MestReNova Version 15.0.1-35756).

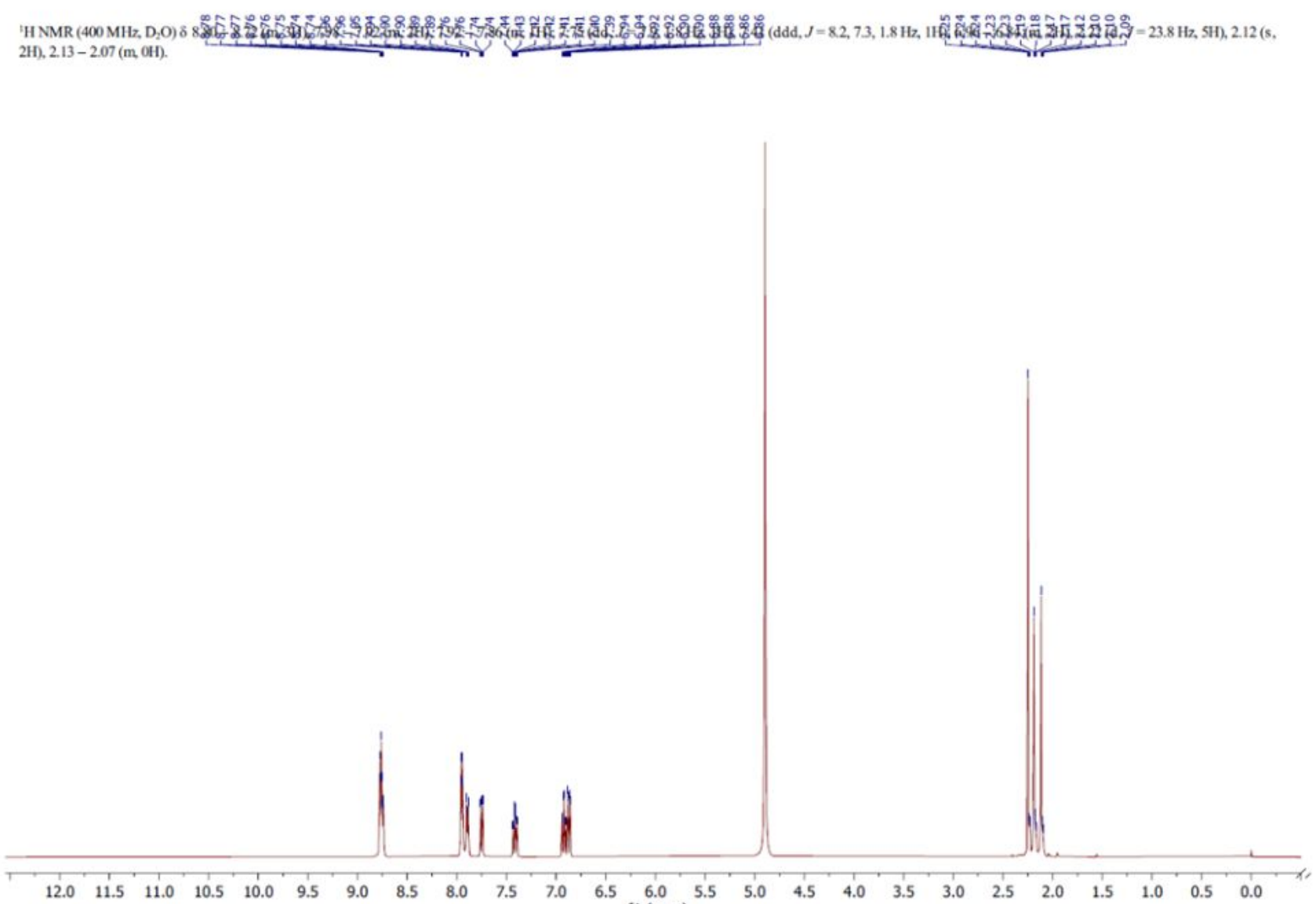

Figure S19: ^{1}H NMR spectra of compound VI recorded in deuterated water at room temperature (MestReNova Version 15.0.1-35756).

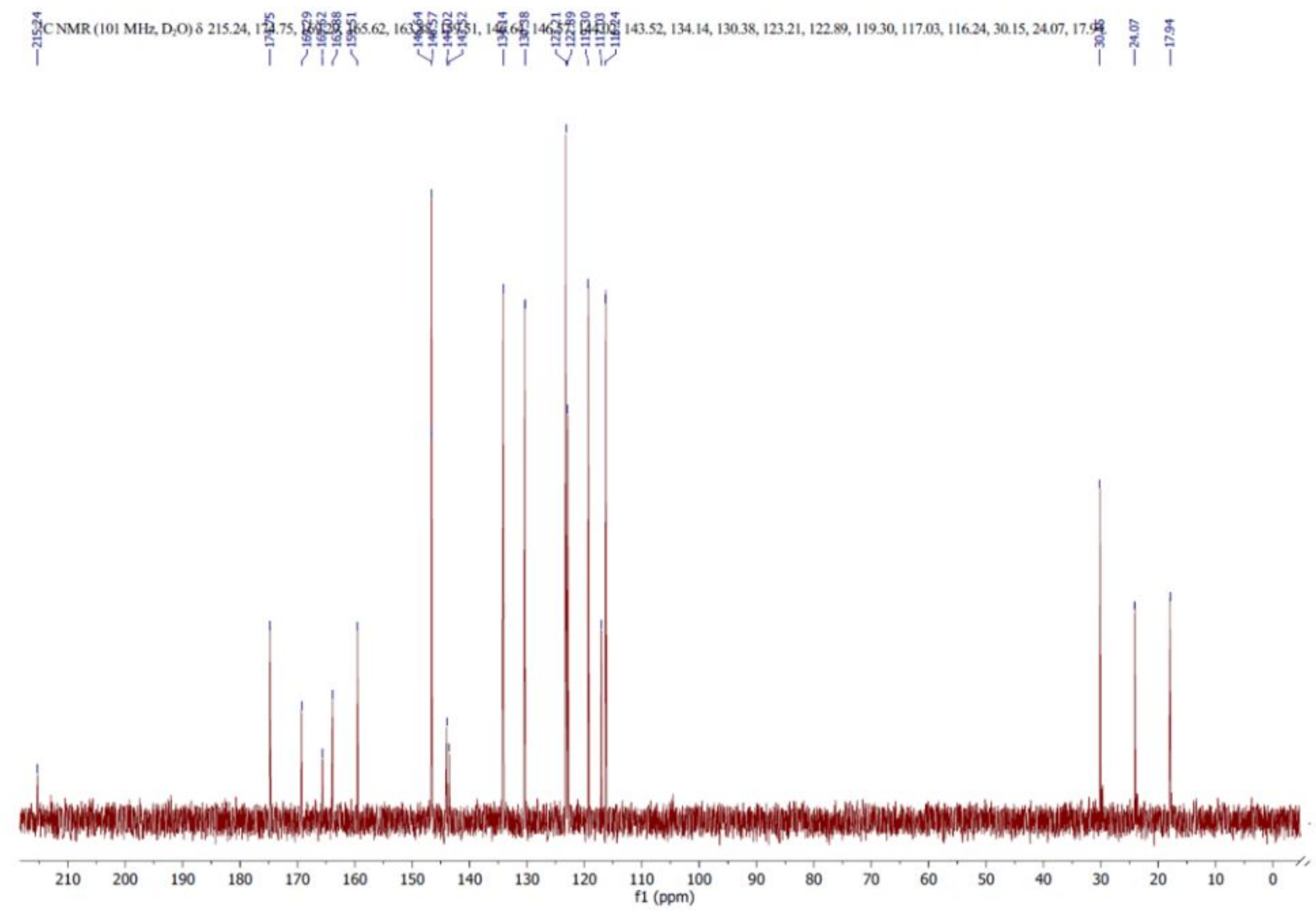

Figure S20: ^{13}C NMR spectra of compound VI recorded in deuterated water at room temperature (MestReNova Version 15.0.1-35756).

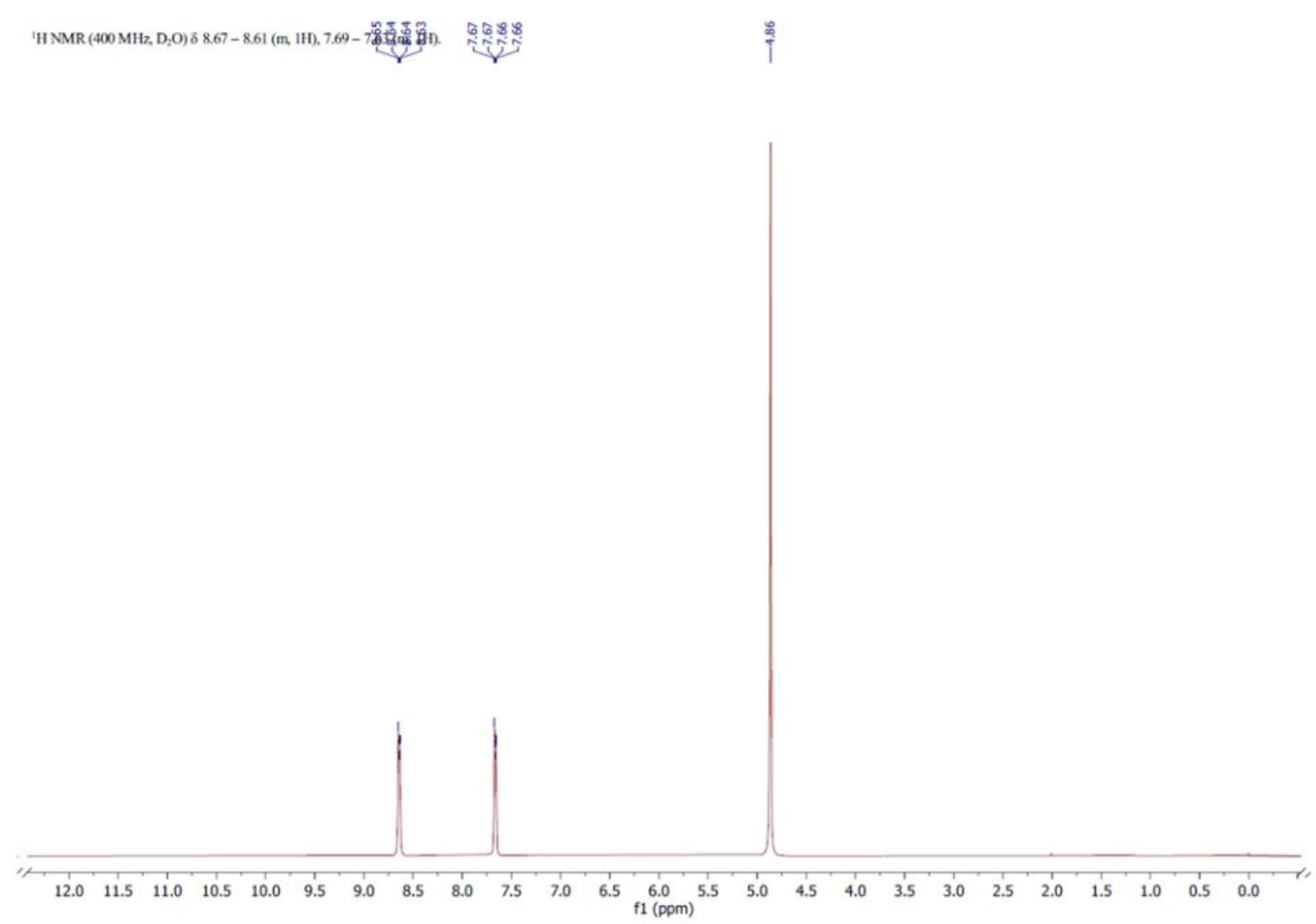

Figure S21: ^{1}H NMR spectra of compound VII recorded in deuterated water at room temperature (MestReNova Version 15.0.1-35756).

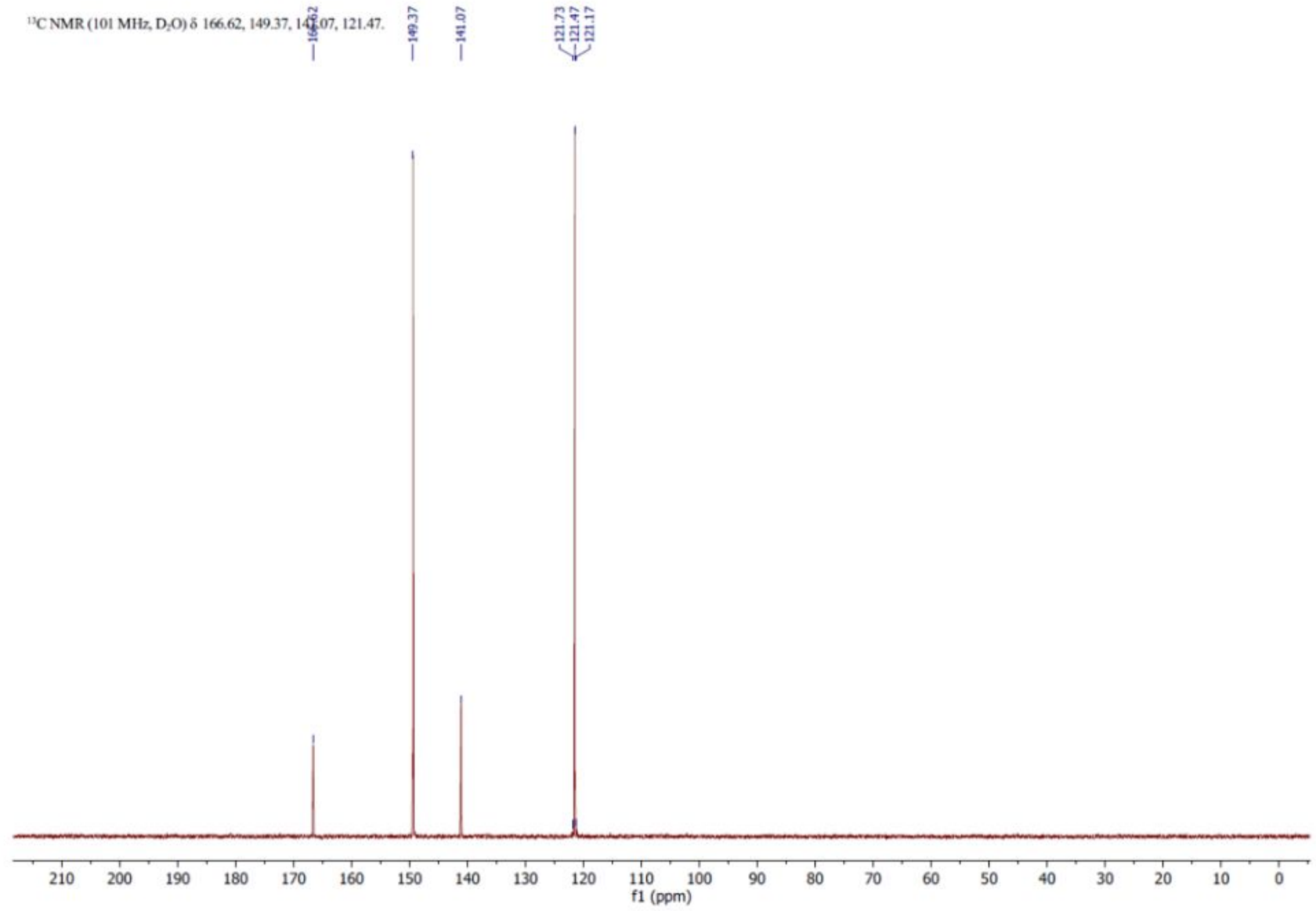

Figure S22: ^{13}C NMR spectra of compound VII recorded in deuterated water at room temperature (MestReNova Version 15.0.1-35756).

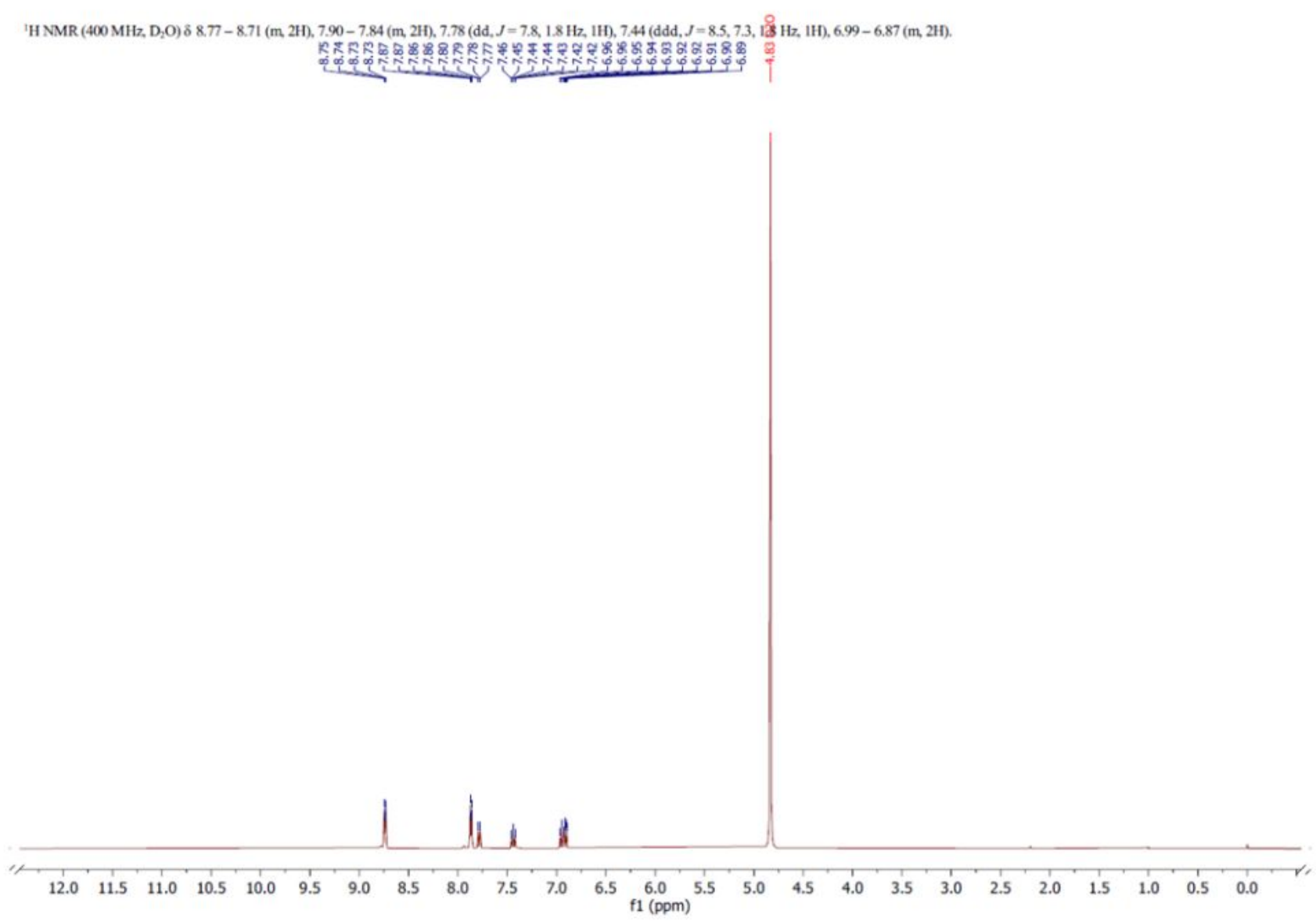

Figure S23: ^{1}H NMR spectra of compound VIII recorded in deuterated water at room temperature (MestReNova Version 15.0.1-35756).

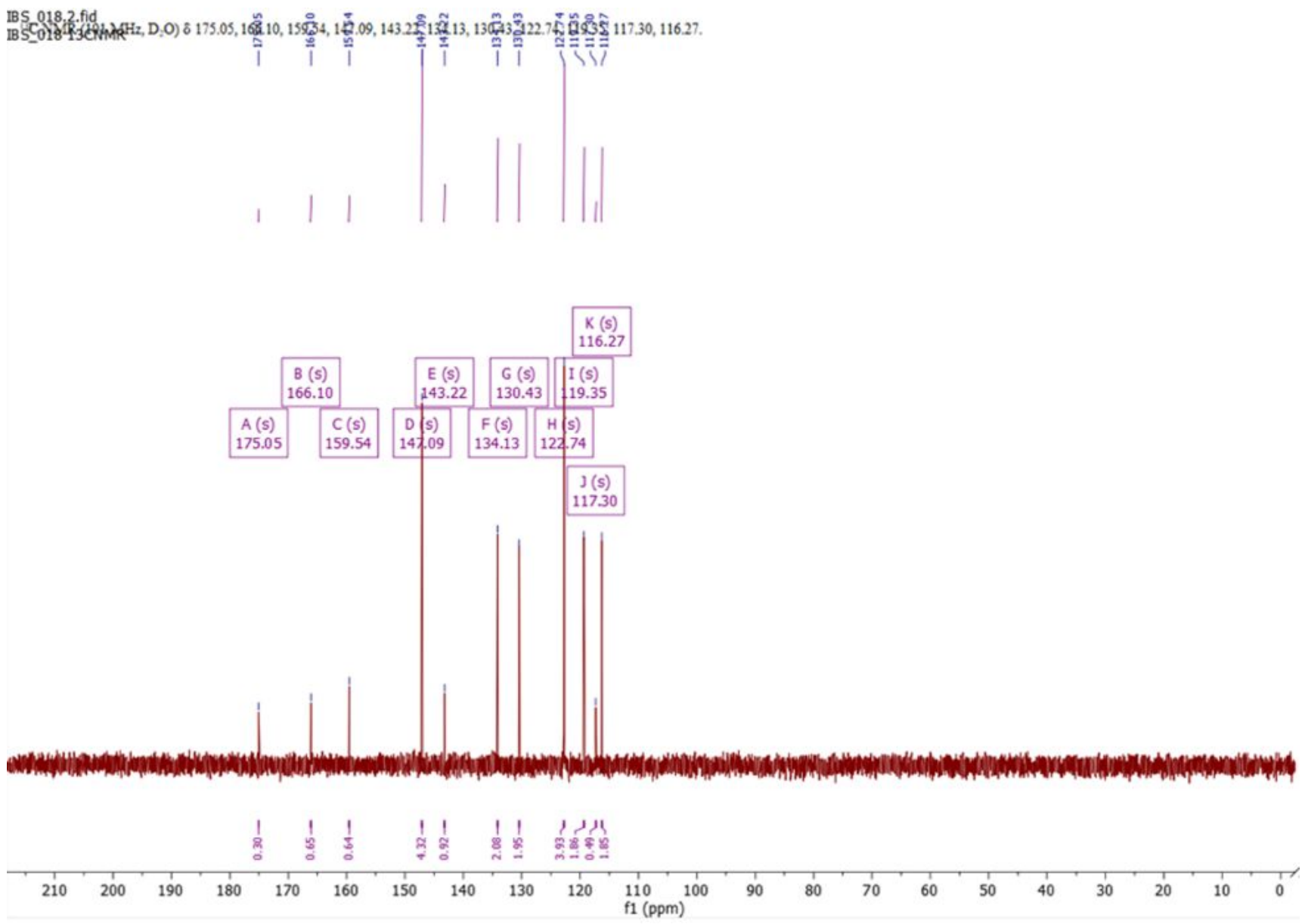

Figure S24: ^{13}C NMR spectra of compound VIII recorded in deuterated water at room temperature (MestReNova Version 15.0.1-35756).

Table S4: Quantitative distribution of flow cytometry populations for *Candida* spp. following treatment with modified isoniazid derivatives. Population percentages were determined from PI-stained cells using standardised gating conditions and interpreted comparatively across treatment groups.

Treatment	PI^+ / PI^- (%)	PI^+ / PI^+ (%)	PI^- / PI^- (%)	PI^- / PI^+ (%)
Candida auris				
Ethanol (50%)	2.9	85.6	2.1	9.4
Compound I	9.5	59.2	20.0	11.3
Compound II	1.6	96.1	0.9	1.4
Compound III	23.4	75.3	0.9	0.4
Compound V	1.3	93.8	0.8	4.2

Compound VI	0.8	97.2	0.3	1.7
Compound VII	5.0	87.7	5.1	2.2
Compound VIII	14.7	64.3	12.0	9.0
Amphotericin B	42.7	57.2	0.1	0.0
Candida glabrata				
Ethanol (50%)	1.5	98.2	0.2	0.2
Compound I	45.5	47.0	3.5	4.1
Compound II	4.0	94.6		
Compound III	23.0	76.1	0.9	0.0
Amphotericin B	40.0	60.0	0.0	0.0
Candida parapsilosis				
Ethanol (50%)	0.1	98.5	0.0	1.4
Compound II	4.2	93.1	1.9	0.8
Compound V	2.9	94.6	1.4	1.2
Compound VI	4.1	91.6	2.6	1.7
Compound VII	3.0	94.0	2.4	0.6
Compound VIII	3.6	93.0	2.9	0.5
Amphotericin B	42.0	57.9	0.1	0.0
